# Supplementary material for: YAP-dependent necrosis occurs in early stages of Alzheimer’s disease and regulates mouse model pathology
Source: Nat Commun. 2020 Jan 24;11:507. doi: 10.1038/s41467-020-14353-6 (PMC6981281; doi:10.1038/s41467-020-14353-6)
Supplement: Supplementary file 2 — Supplementary Information [file 41467_2020_14353_MOESM2_ESM.pdf]

Supplementary Information

YAP-dependent necrosis occurs in early stages of Alzheimer's disease and regulates mouse model pathology

Tanaka et al.

Supplementary Figure 1

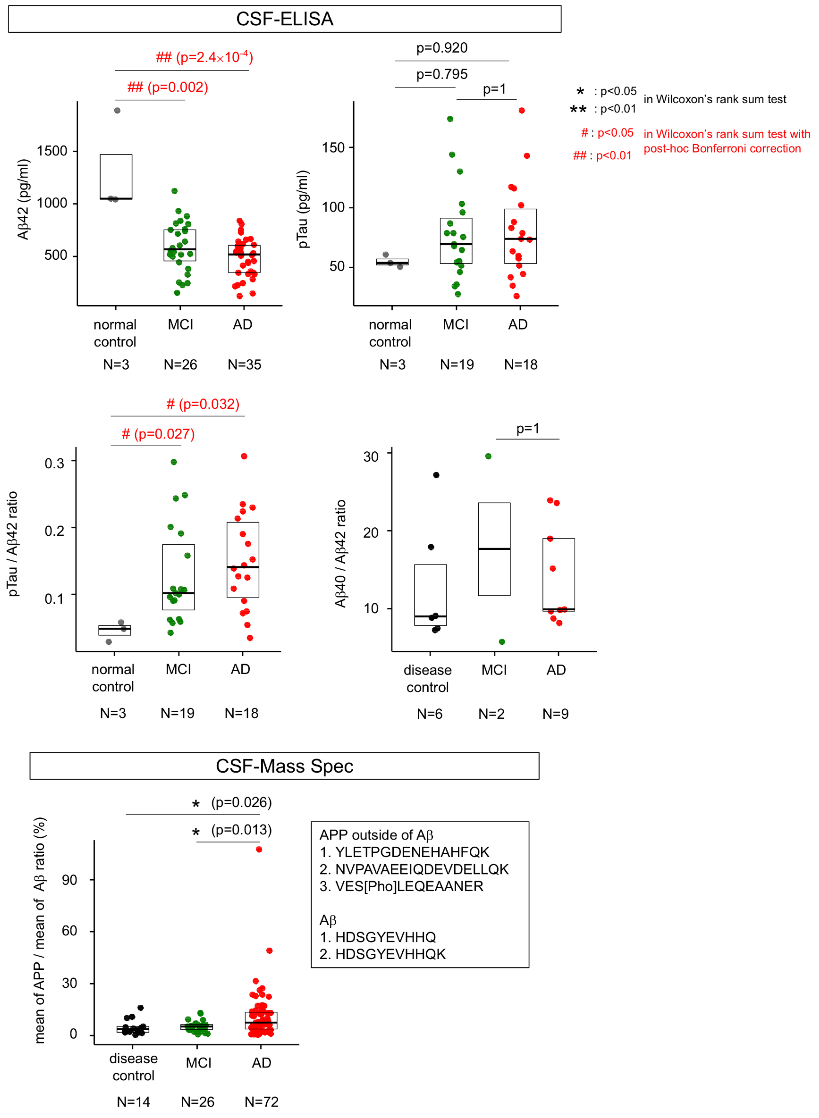

Supplementary Figure 1

CSF-Aβ_{42} and CSF-pTau in MCI/AD patients

Levels of CSF-Aβ42, CSF-pTau, the CSF-Aβ40/CSF-Aβ42 ratio and the CSF-pTau/CSF-Aβ42 ratio in CSF of the normal control, MCI, and AD groups were evaluated by ELISA. Box plots show the median and quartiles. Statistical differences among groups were evaluated using the Wilcoxon rank–sum test with post-hoc Bonferroni correction, #$p<0.05$, ##$p<0.01$. Reduction in the CSF-Aβ42 level and an increase in the CSF-pTau/CSF-Aβ42 ratio in the MCI/AD groups verified the clinical diagnoses of patients in this study.

Levels of the APP/Aβ ratio in CSF of the normal control, MCI, and AD groups were evaluated by mass spectrometry. Amounts of 5 peptides from APP protein were determined by the SWATH analysis, and 2 of 5 peptides were overlapped on the sequence of Aβ and the other three were derived from APP outside of Aβ. Ratio between the mean value of two types of Aβ peptides in CSF samples and the mean value of three non-Aβ APP peptides was calculated in each sample, and plotted on to the graph. The box plot shows the median and quartiles. Statistical differences among groups were evaluated using the Wilcoxon rank–sum test, *$p<0.05$ (N: shown below graphs).

Source data are provided as a “Source Data file”.

Supplementary Figure 2

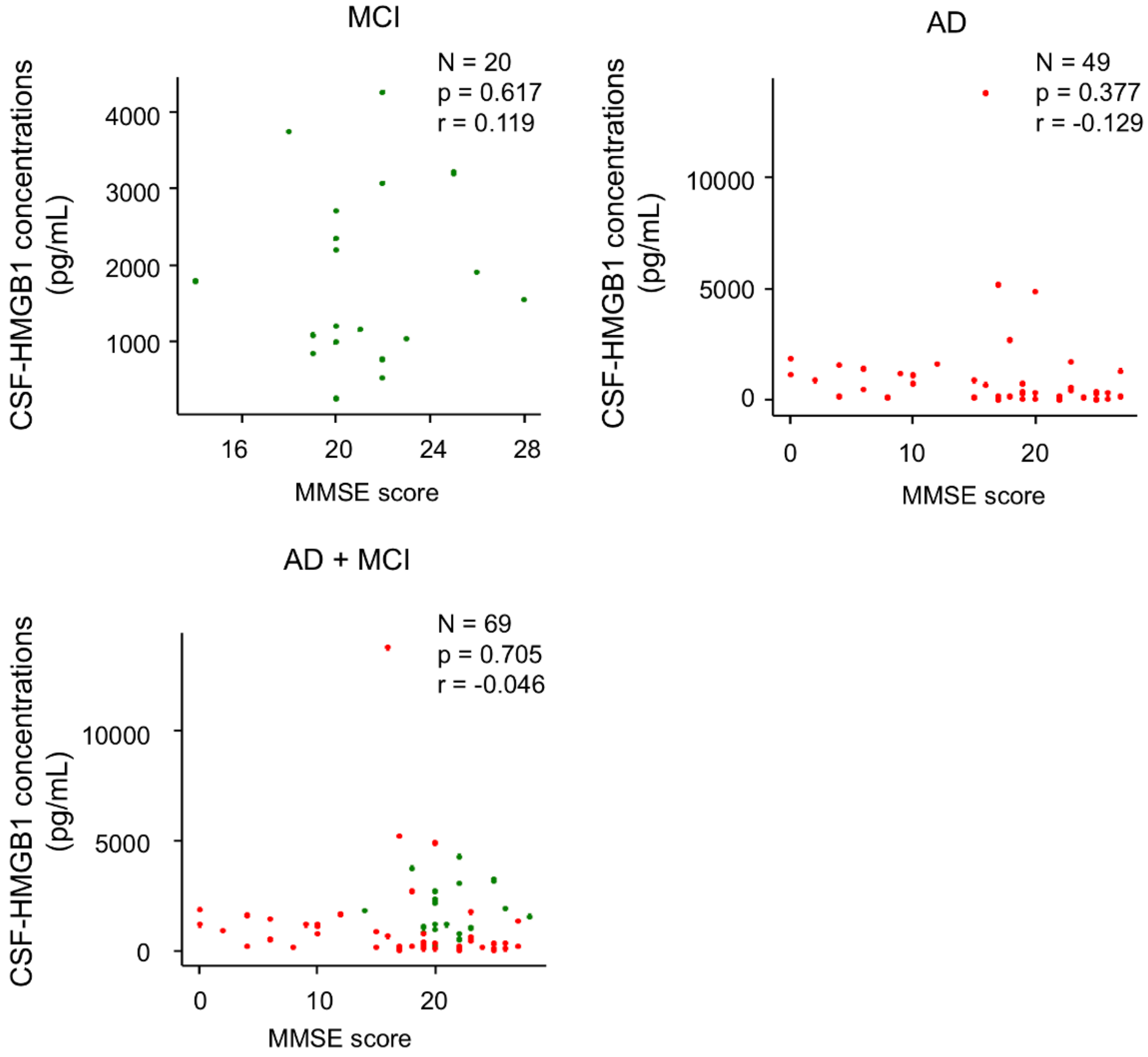

Supplementary Figure 2

No correlation between CSF-HMGB1 concentration and MMSE scores in the MCI and/or AD group

Correlations between CSF-HMGB1 levels and MMSE scores for the MCI, AD, and MCI+AD groups. P-values were determined by Pearson's correlation coefficient (N: shown in graphs).

Source data are provided as a "Source Data file".

Supplementary Figure 3

r, p : Pearson's correlation coefficient and p-value

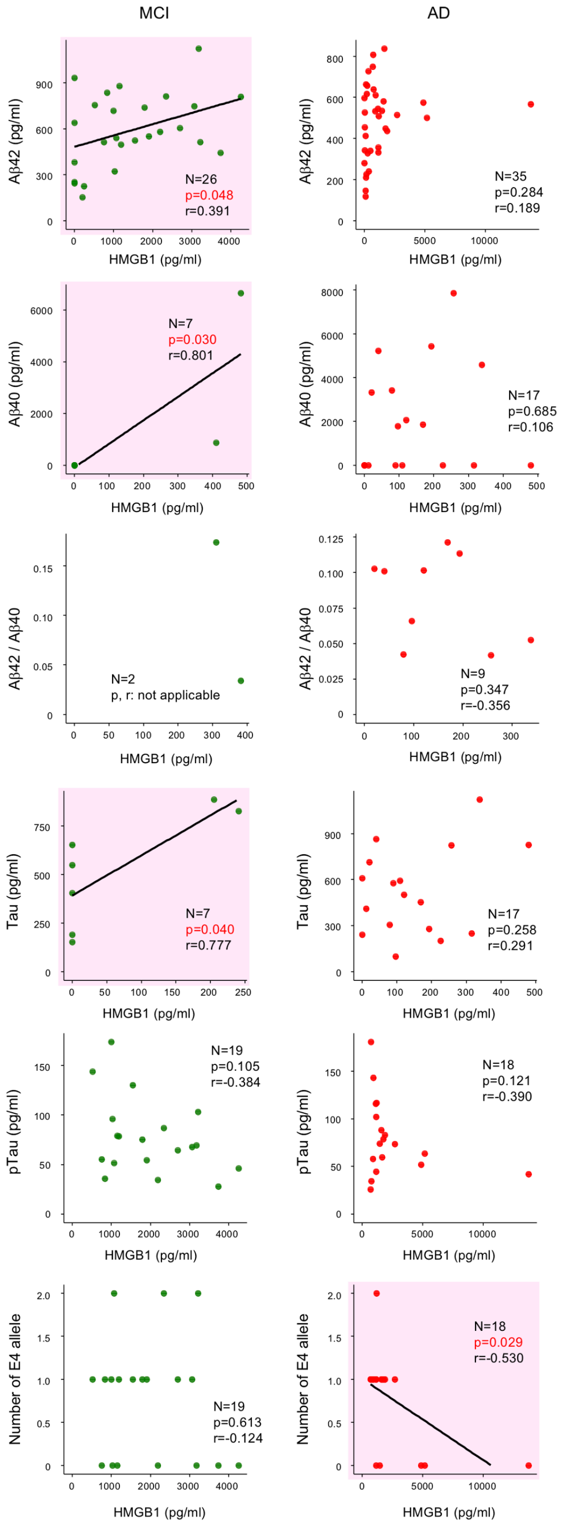

Supplementary Figure 3

Relationship between CSF-HMGB1 levels and other CSF biomarkers

Correlations between CSF-HMGB1 levels and the biomarkers Aβ42, Aβ40, tau, pTau, and *APOE4* allele copy number are shown. Colors indicate cases in which the correlation of paired parameters was statistically confirmed. P-values were determined by Pearson's correlation coefficient (N: shown in graphs).

Source data are provided as a "Source Data file".

Supplementary Figure 4

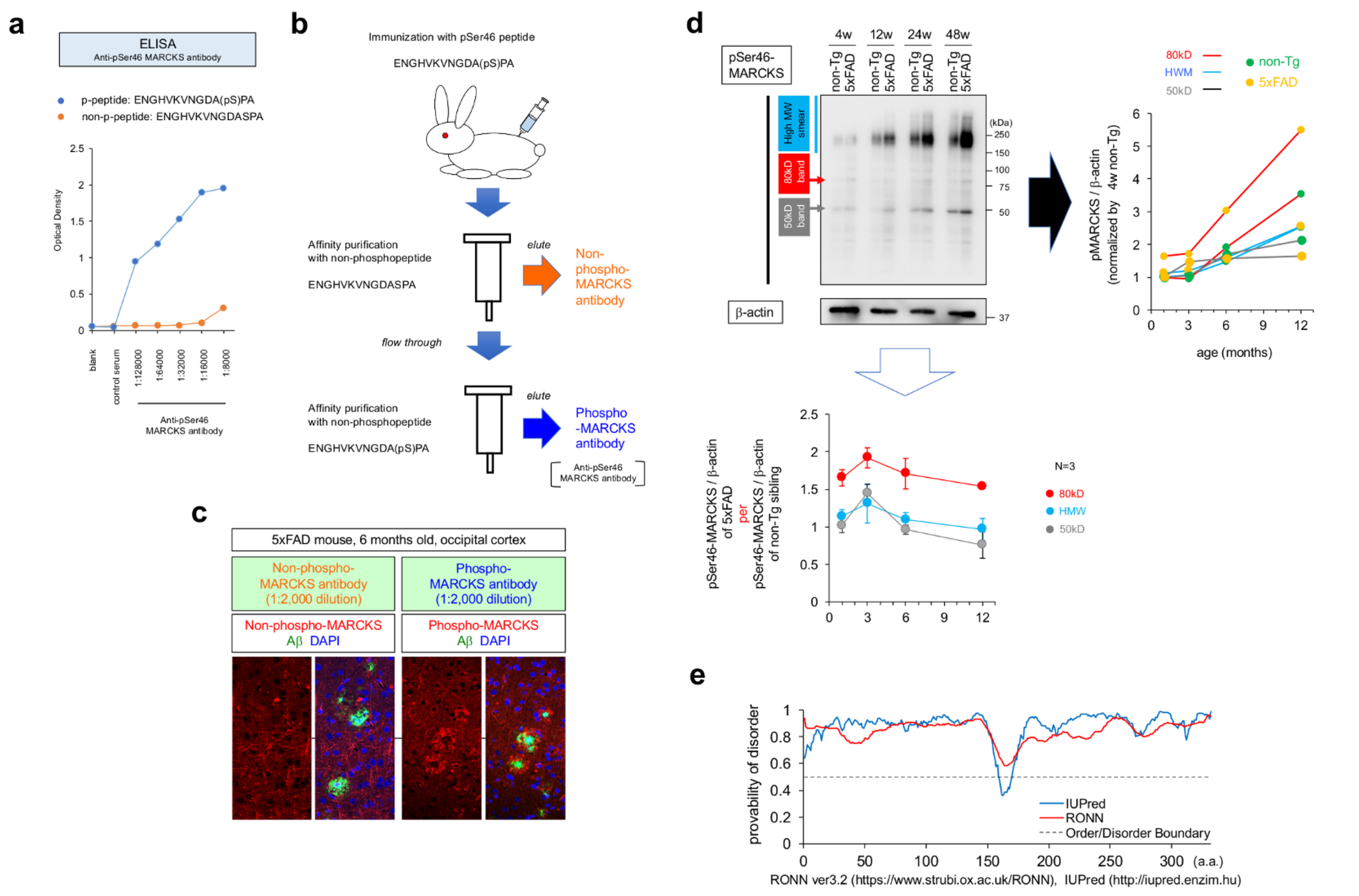

Supplementary Figure 4

a. Reactivity of anti-pSer46-MARCKS antibody to phosphorylated and non-phosphorylated peptides of MARCKS in ELISA.

b. Protocol for generation of anti-pSer46-MARCKS antibody.

c. Anti-pSer46-MARCKS and anti-non-phosphorylated MARCKS antibodies differentially stained structures around extracellular Aβ plaques in immunohistochemistry.

d. Western blot of pSer46-MARCKS proteins during normal and abnormal aging. Right graph shows the chronological change of signals of high molecular weight (HMW) smear, 80kD band and 50kD band during aging of 5xFAD and non-transgenic sibling mice from 1 to 12 months. Lower graph shoes the ratio of pSer46-MARCKS between 5xFAD and non-transgenic sibling mice at each time point. Values in each group are summarized by mean ± S.E.M. (N=3 mice).

e. Probability of disordered structure in MARCKS protein by two prediction methods. The URLs are shown under the graph.

Source data are provided as a “Source Data file”.

Supplementary Figure 5

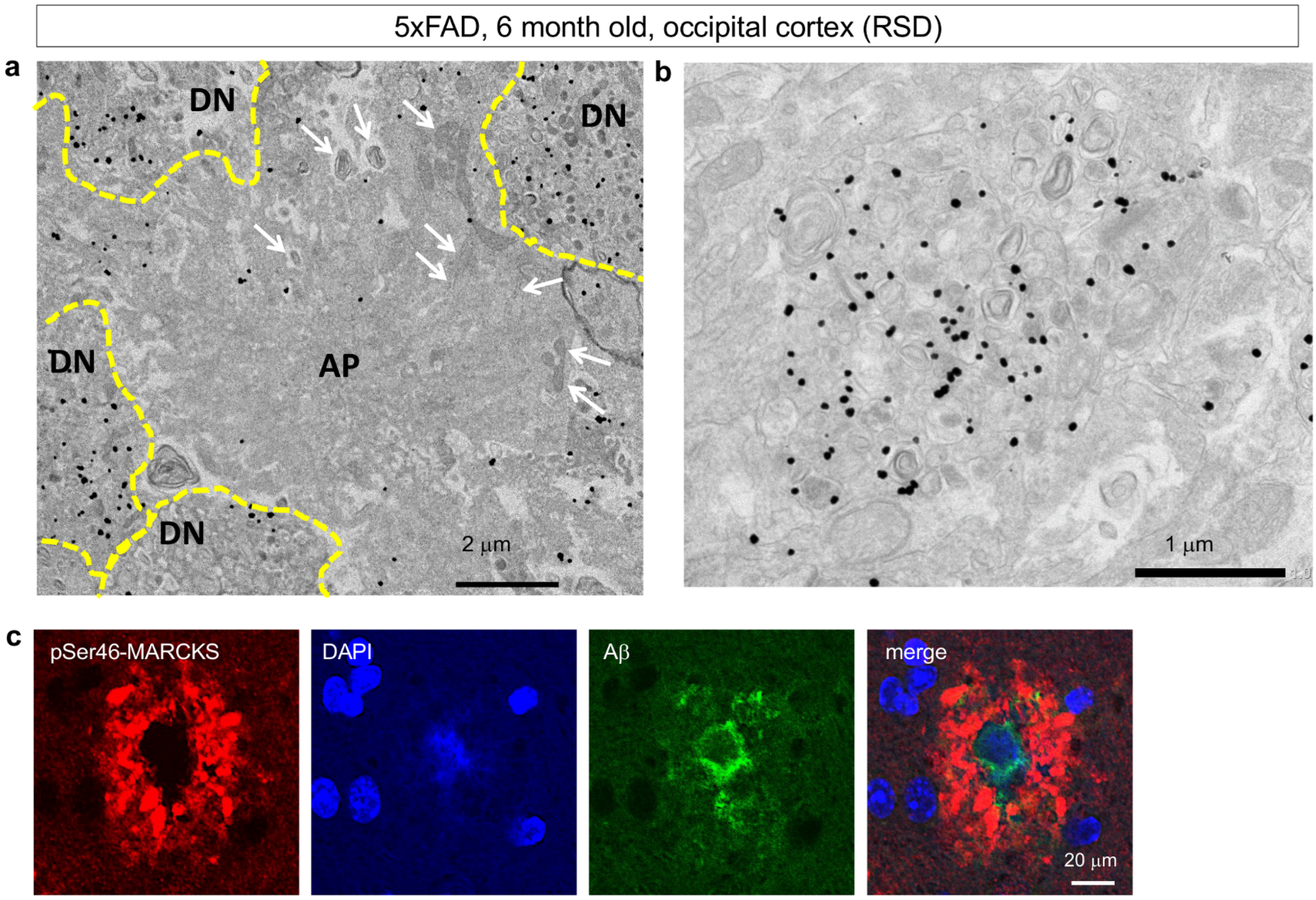

Supplementary Figure 5

Immunoelectron microscopy of degenerative neurites and amyloid plaques

a. Cerebral cortex tissues of 5xFAD mice at 6 months of age were examined by immunoelectron microscopy. Degenerative neurites (**DN**) surrounding amyloid plaques (**AP**) showed strong reactivity to anti-Ser46-MARCKS antibody (areas in yellow dot lines). Even in the core of extracellular amyloid depositions, organelles (white arrows) like mitochondria, ER and phagosome were present, indicating that these areas represented the remnants of ruined cell(s) that had presumably contained intracellular Aβ.

b. Degenerative neurites included numerous phagosomes with double-membrane structures.

c. Confocal microscopy image of amyloid plaques triple-stained by pSer46-MARCKS (red), Aβ (green), and DAPI (blue) in cerebral cortex of 5xFAD mice at 3 months of age. Consistent with (**a**), residual nuclear DNA components were stained by DAPI in the center of intracellular Aβ and extracellular Aβ that began to aggregate. Reactive phosphorylation of MARCKS at Ser46 was stained in degenerative neurites surrounding the early-stage extracellular Aβ plaques. Similar images were reported in human PS1-linked AD and APP-KI mice previously by our group[22].

Supplementary Figure 6

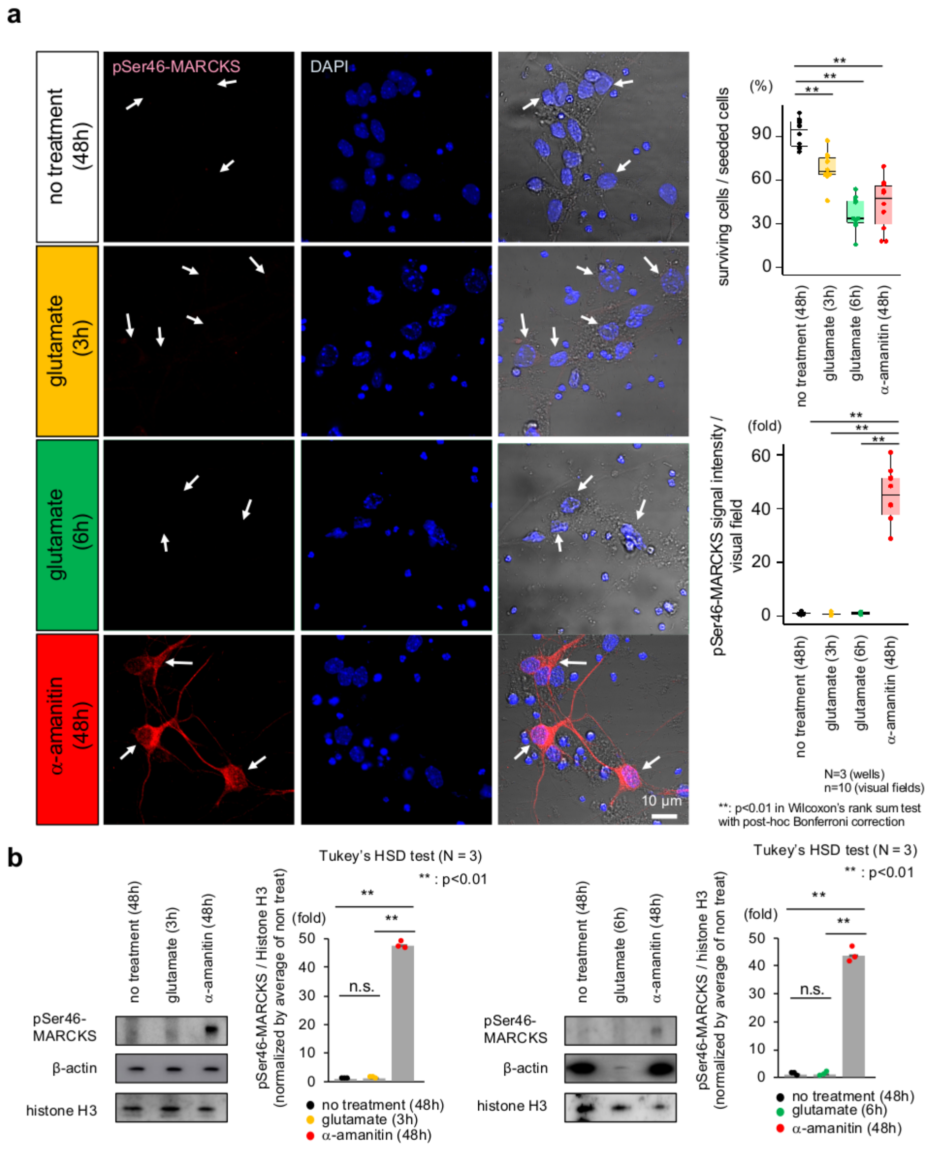

Supplementary Figure 6

Necrosis but not apoptosis induces pSer46-MARCKS

a. Immunocytochemistry of primary mouse cortical neurons under glutamate-induced apoptosis or α-amanitin-induced necrosis by using anti-pSer46-MARCKS antibody. pSer46-MARCKS was induced in surviving neurons under necrosis (arrow) but in surviving neurons under apoptosis (arrow). The conditions for inducing apoptosis and necrosis were described in details in Methods. Right upper graph shows the percentage of surviving cells in each condition. Right lower graph shows signal intensities of pSer46-MARCKS per visual field. The signal intensities (a.u.) in each condition were shown as the ratio normalized by the value of non-treated condition. DIC: differential interference contrast. Box plots show the median, quartiles and whiskers that represent 1.5× the interquartile range. P-values were determined by Wilcoxon's rank sum test with post-hoc Bonferroni correction, **p<0.01 (N=3 wells, n=10 visual fields).

b. Western blot confirmed the induction of pSer46-MARCKS in surviving neurons under α-amanitin-induced necrosis but not under glutamate-induced apoptosis. Right graphs show signal intensities of pSer46-MARCKS band in each condition normalized by the background signal. The signal intensities (a.u.) in each condition were shown as the ratio normalized by the value of non-treated condition. Some control genes such as β-actin was reduced even at the time point when some neurons still kept the morphology (6 hours after addition of glutamate) but histone H3 was not changed at the same time point. pSer46-MARCKS was not induced under apoptosis even at 3 hours after addition of glutamate when the percentage of surviving cells was higher than that under necrosis at 48 hours after addition of α-amanitin. The bar graph indicates average and mean ± S.E.M., together with the corresponding data points. P-values were determined by Tukey's HSD test, $^{**}p<0.01$ (N=3 tests).

Source data are provided as a "Source Data file".

Supplementary Figure 7

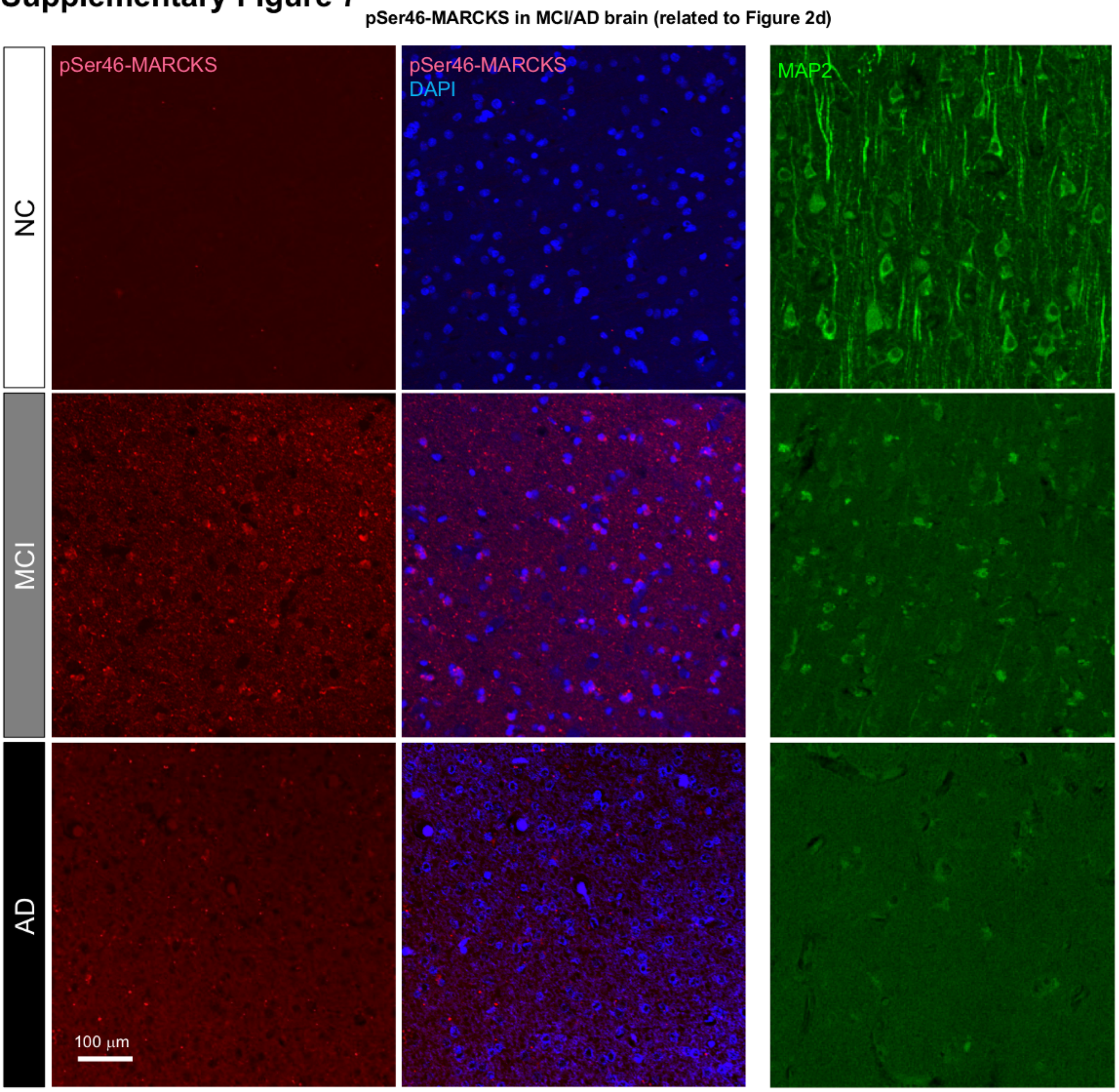

Supplementary Figure 7

Neurons associated with reactive pSer46-MARCKS stains in human MCI

Immunostaining of cerebral cortex tissues (temporal tips) of MCI/AD patients with anti-pSer46-MARCKS antibody. DAPI-positive cells were frequently accompanied with pSer46-MARCKS stains in MCI patients (MCI) but not in non-neurological disease controls (NC) or AD patients (AD). In AD patients, the number of neurons by itself was remarkably decreased.

Supplementary Figure 8

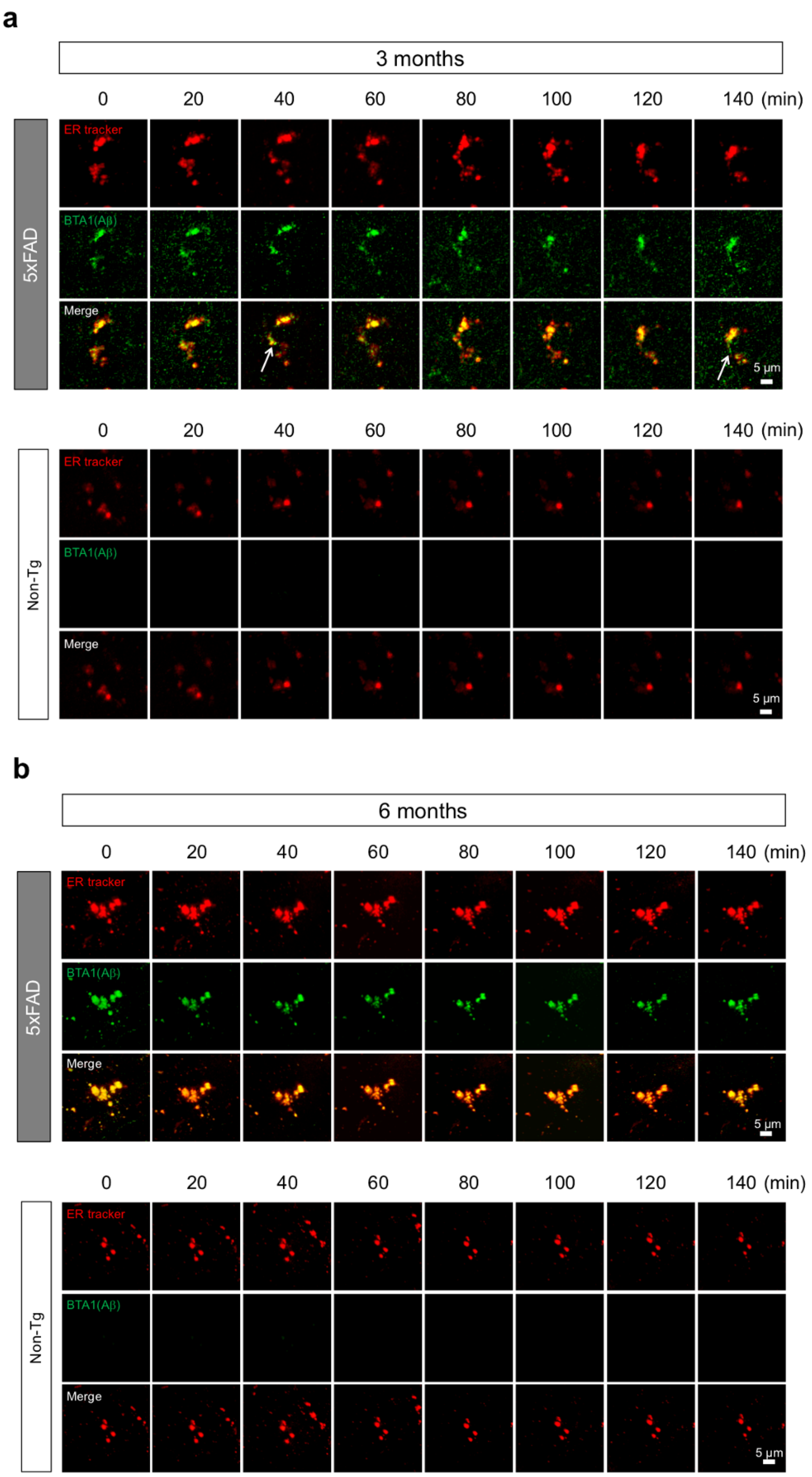

Supplementary Figure 8

In vivo ER imaging of cortical neurons of 5xFAD mice at 3 months of age

Expansion and instability of ER was observed at 3 months of age **(a)** and 6 months of age **(b)**, similarly to 1 month. Intracellular Aβ stained by BTA1 was mostly localized to ER, although a small portion leaked out into the cytoplasm (arrow).

Supplementary Figure 9

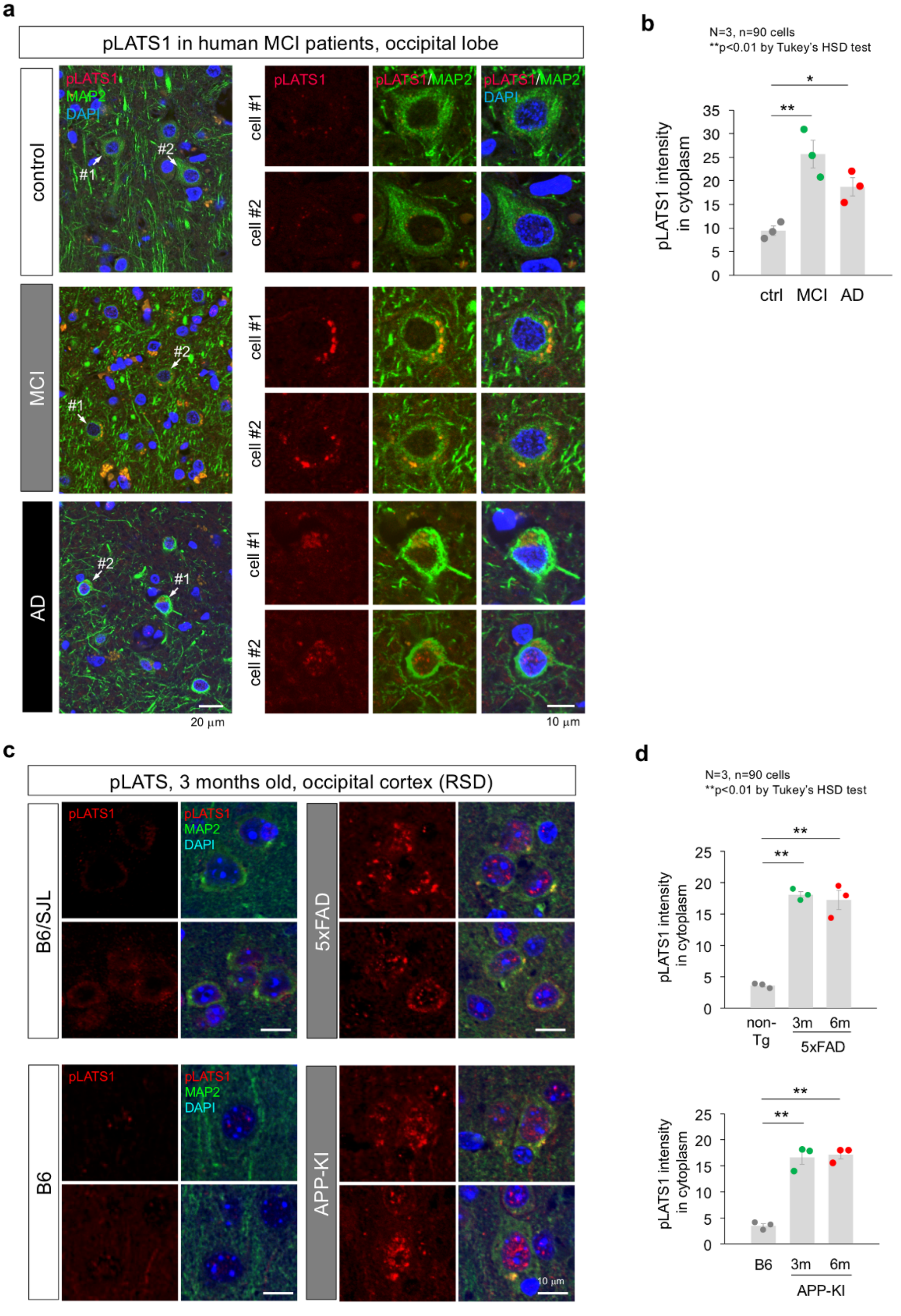

Supplementary Figure 9

LATS1 activation in cortical neurons of human MCI and AD patients

a, b. Double staining of MAP2 and phospho-LATS1 (Ser909) confirmed that LATS1 kinase was activated in neurons at MCI stage, although activation was weakened at the symptomatic AD stage. Signal intensities of phospho-LATS1 in neuronal cytoplasm were quantitatively analyzed in three groups (lower graph). The bar graph indicates average and mean ± S.E.M., together with the corresponding data points. P-values were determined by Tukey's HSD test, **p<0.01 (N=3 persons, n=90 cells).

c. Double staining of occipital cortex tissues of 5xFAD mice with anti-MAP2 and phospho-LATS1 (Ser909) antibodies.

d. Signal intensities of pSer909-LATS1 in neurons were quantitatively compared between non-Tg sibling (3 months) and 5xFAD mice (3 months and 6 months). The bar graph indicates average and mean ± S.E.M., together with the corresponding data points. P-values were determined by Tukey's HSD test, $^{**}p<0.01$ (N=3 mice, n=90 cells).

Source data are provided as a "Source Data file".

Supplementary Figure 10

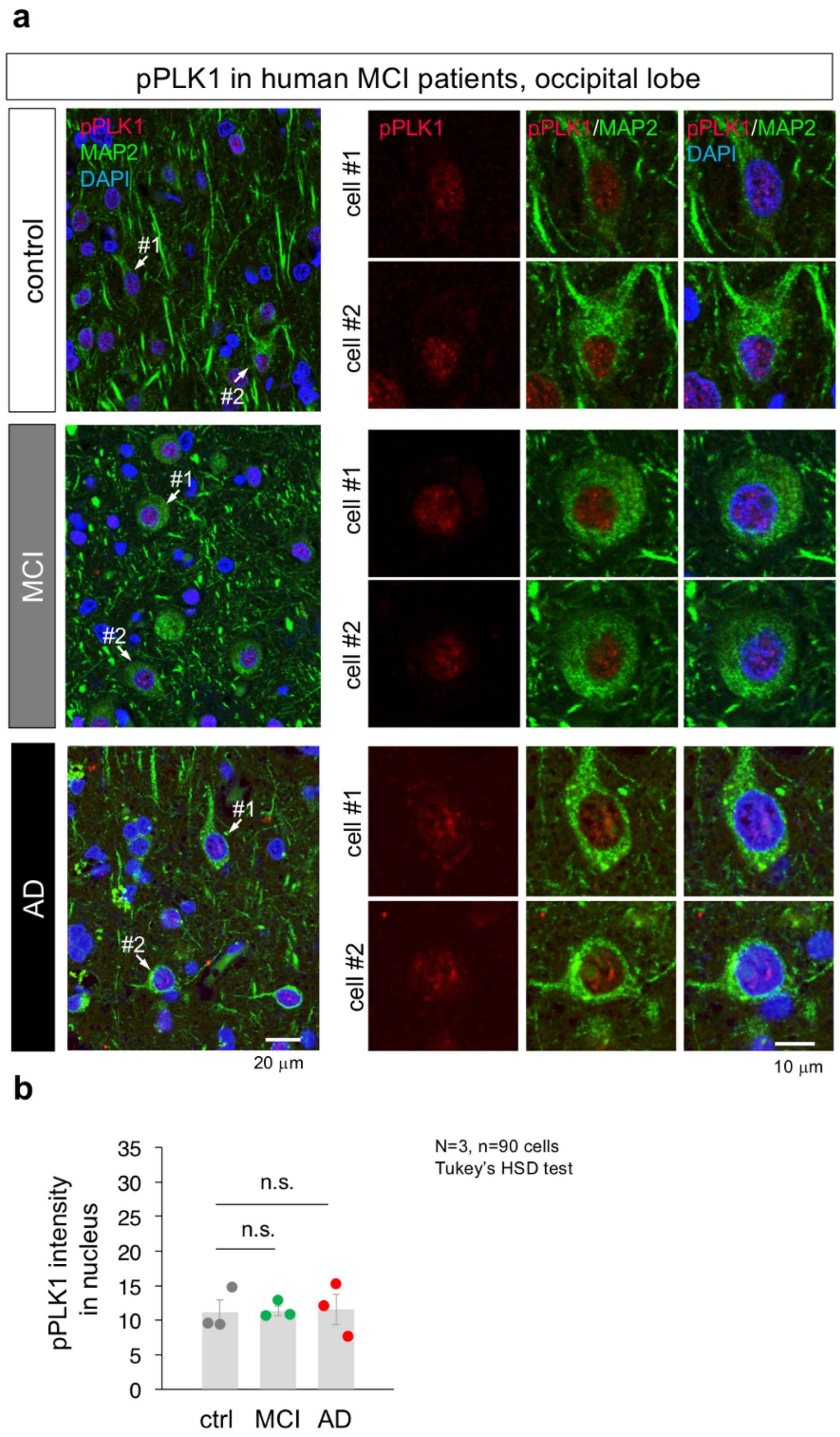

Supplementary Figure 10

Plk1 inactivation in cortical neurons of human MCI and AD patients

a, b. Double staining of MAP2 and phospho-Plk1 (pThr210) confirmed that Plk1 kinase was not activated in neurons at the MCI or AD symptomatic stage. Signal intensities of phospho-Plk1 in neuronal nucleus were quantitatively analyzed in three groups (lower graph). The bar graph indicates average and mean ± S.E.M., together with the corresponding data points. P-values were determined by Tukey's HSD test (N=3 persons, n=90 cells).

Source data are provided as a "Source Data file".

Supplementary Figure 11

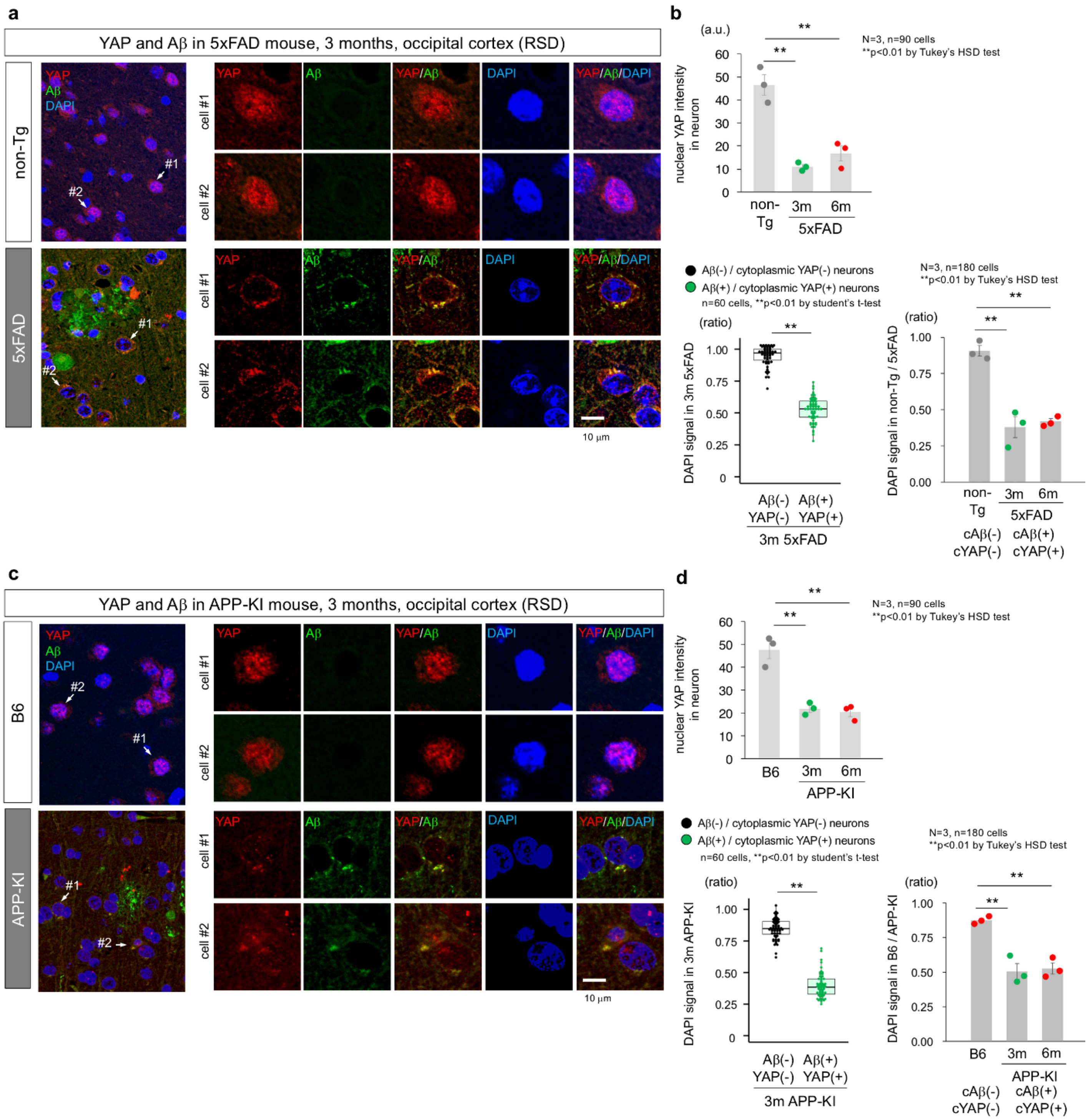

Supplementary Figure 11

Sequestration of YAP to cytoplasmic Aβ aggregates in cortical neurons of AD mouse models

a. Immunohistochemistry of YAP and Aβ of 5xFAD mouse brain revealed sequestration of YAP to cytoplasmic Aβ aggregates and a resultant decrease in the level of nuclear YAP.

b. Quantitative analysis of signal intensity of YAP in nucleus. Total nuclear YAP signals were quantified in 90 neurons (N=3 mice) by confocal microscopy (FV1200IXGP44, Olympus, Tokyo, Japan). Three mice in each group were used for the analysis.

Quantitative analysis of signal intensity of DAPI in nucleus. Total nuclear DAPI signals were quantified in cytoplasmic YAP-positive and Aβ-positive neurons (n=60) and in cytoplasmic YAP-negative and Aβ-negative neurons (n=60) of 5xFAD mice. In addition, signal intensity of DAPI per a nucleus was compared between cytoplasmic YAP-positive and Aβ-positive neurons (n=180) of 5xFAD mice at 3 or 6 months (N=3) and normal neurons (n=180) of the non-transgenic sibling mice at 3 months (N=3). Box plots show the median, quartiles and whiskers that represent 1.5× the interquartile range. The bar graph indicates average and mean ± S.E.M., together with the corresponding data points. P-values were determined by Tukey's HSD test or student's t-test, $^{**}p<0.01$.

c. Immunohistochemistry of YAP and Aβ of APP-KI mouse brain revealed sequestration of YAP to cytoplasmic Aβ aggregates and a resultant decrease in the level of nuclear YAP.

d. Quantitative analysis of signal intensity of YAP in nucleus. Total nuclear YAP signals were quantified in 90 neurons (N=3 mice) by confocal microscopy (FV1200IXGP44, Olympus, Tokyo, Japan). Three mice in each group were used for the analysis.

Quantitative analysis of signal intensity of DAPI in nucleus. Total nuclear DAPI signals were quantified in cytoplasmic YAP-positive and Aβ-positive neurons (n=60) and in cytoplasmic YAP-negative and Aβ-negative neurons (n=60) of human mutant APP-KI mice. In addition, signal intensity of DAPI per a nucleus was compared between cytoplasmic YAP-positive and Aβ-positive neurons (n=180) of human mutant APP-KI mice at 3 or 6 months (N=3) and normal neurons (n=180) of the non-transgenic sibling mice at 3 months (N=3). Box plots show the median, quartiles and whiskers that represent 1.5× the interquartile range. The bar graph indicates average and mean ± S.E.M., together with the corresponding data points. P-values were determined by Tukey's HSD test or student's t-test, $^{**}p<0.01$.

Source data are provided as a "Source Data file".

Supplementary Figure 12

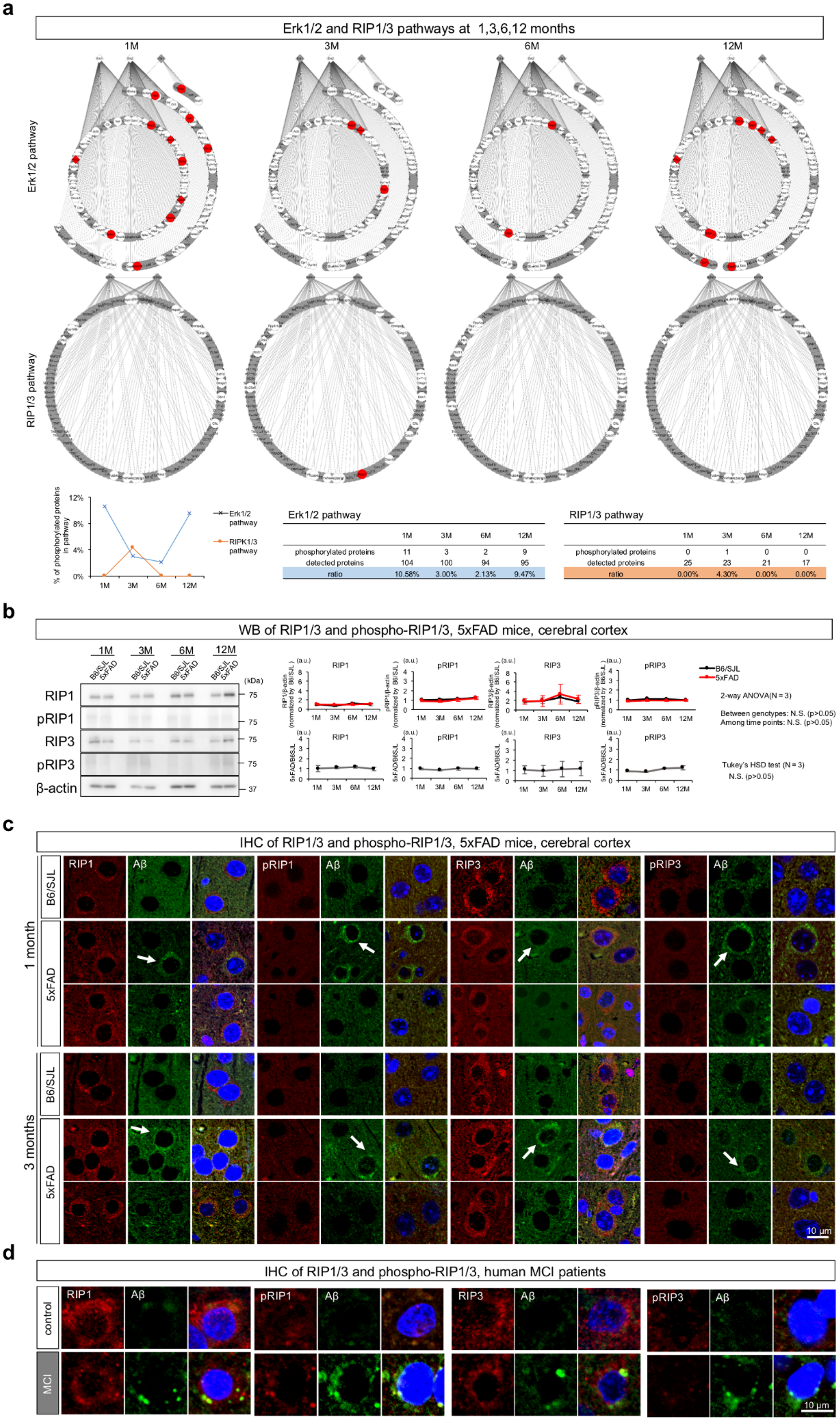

Erk1/2 pathway

	1M	3M	6M	12M
phosphorylated proteins	11	3	2	9
detected proteins	104	100	94	95
ratio	10.58%	3.00%	2.13%	9.47%

RIP1/3 pathway

	1M	3M	6M	12M
phosphorylated proteins	0	1	0	0
detected proteins	25	23	21	17
ratio	0.00%	4.30%	0.00%	0.00%

Supplementary Figure 12

Activities of Erk1/2 and RIP1/3 signaling pathways in mouse and human

a. Phosphorylation changes of target proteins of Erk1/2 or RIP1/3 revealed by phosphoproteome analysis of whole cerebral tissues from 5xFAD mice. Red nodes indicate increased phosphorylation of the Erk1/2 downstream proteins in comparison to B6/SJL mice. On the other hand, phosphorylation is hardly changed in the downstream proteins of RIP1/3.

b. Western blot of RIP1/3 with whole cerebral cortex tissues from 5xFAD mice and B6/SJL mice at 1 to 12 months of ages. The graph shows the ratio of band intensities 5xFAD mice to B6/SJL mice. Values in each group are summarized by mean ± S.E.M. P-values were determined by 2-way ANOVA or Tukey's HSD test, N.S. $p>0.05$ (N=3 tests).

c. Immunohistochemistry of parietal cortex tissues from 5xFAD and B6/SJL mice at 1 and 3 months of age. Co-staining of RIP1/3 or phospho-RIP1/3 with Aβ (82E1) shows the similar levels of RIP1/3 or phospho-RIP1/3 in intracellular amyloid-positive (arrow) and -negative (no arrow) cells.

d. Immunohistochemistry of cerebral cortex tissues from patients of MCI due to AD and non-neurological disease with anti-Aβ (82E1) and anti-RIP1/3 or anti-phospho-RIP1/3 antibodies.

Source data are provided as a "Source Data file".

Supplementary Figure 13

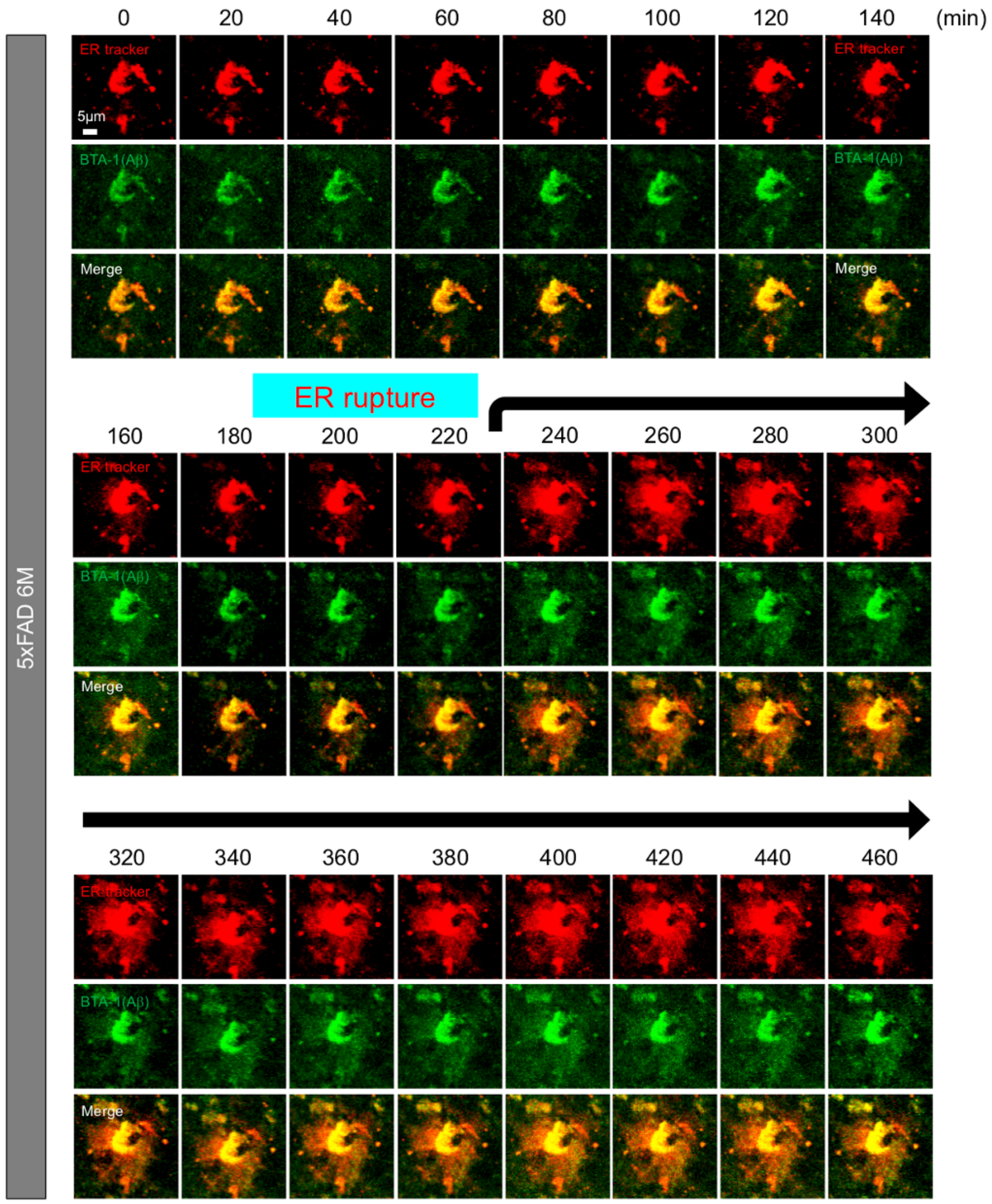

Supplementary Figure 13

***In vivo* ER imaging of 5xFAD mice**

Timelapse imaging of a BTA-stained neuron in cerebral cortex (retrosplenial dysgranular cortex) of 5xFAD mouse at 6 months of age revealed that intracellular Aβ retained in the extracellular space after the rupture of ER (from 240 min of observation) during TRIAD necrosis of the neuron and could be a seed for extracellular Aβ aggregation.

Supplementary Figure 14

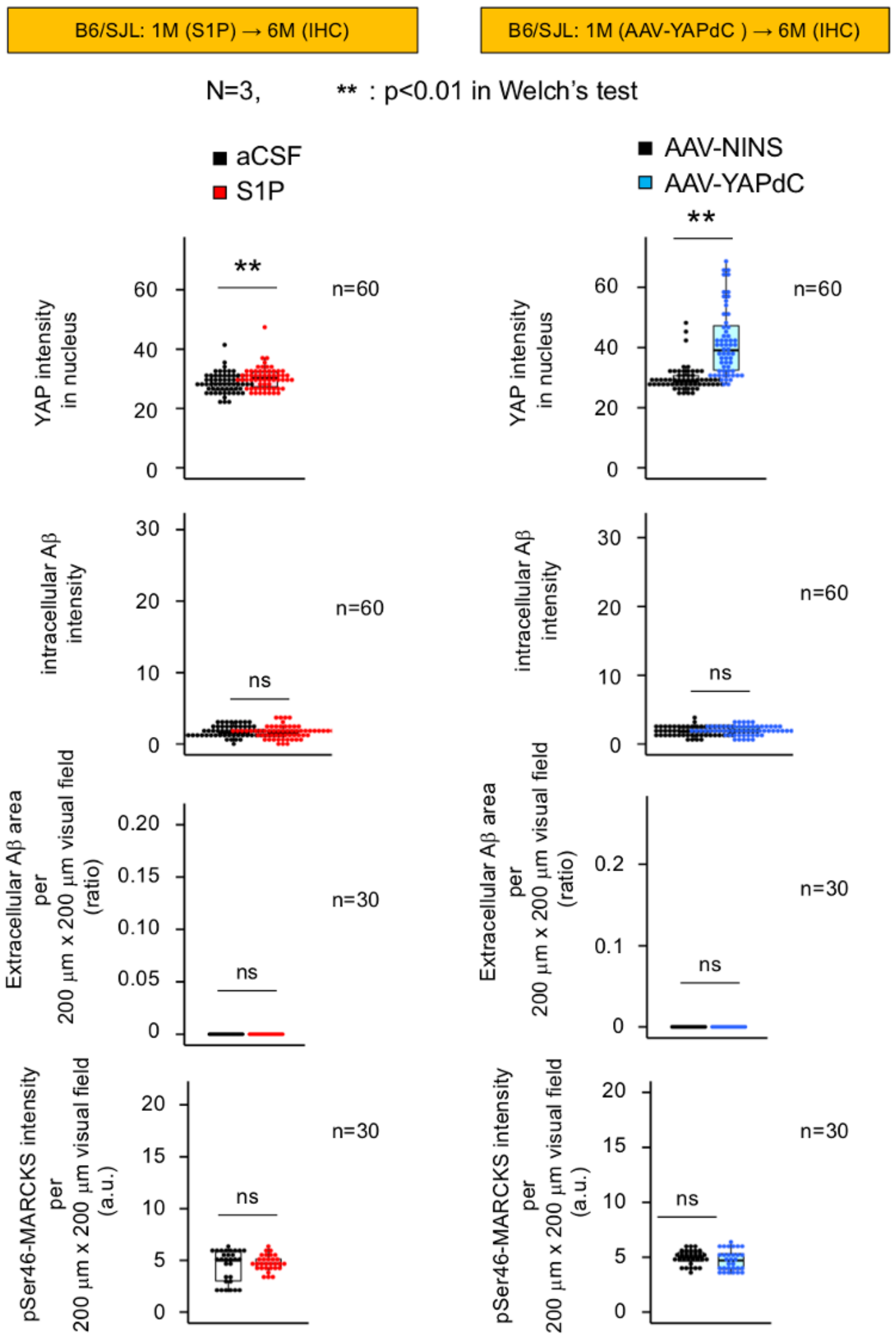

Supplementary Figure 14

Effect of S1P and AAV-YAPdeltaC on normal mice

Normal sibling mice (B6/SJL) that had been treated similarly as 5xFAD mice in Figure 9 were examined for total YAP intensity, intracellular Aβ, extracellular Aβ, and pSer46-MARCKS. Box plots show the median, quartiles and whiskers that represent 1.5× the interquartile range. P-values were determined by Welch's test, **$p<0.01$. (N=3 mice, n=30 visual fields or 60 cells)

Source data are provided as a "Source Data file".

Supplementary Figure 15

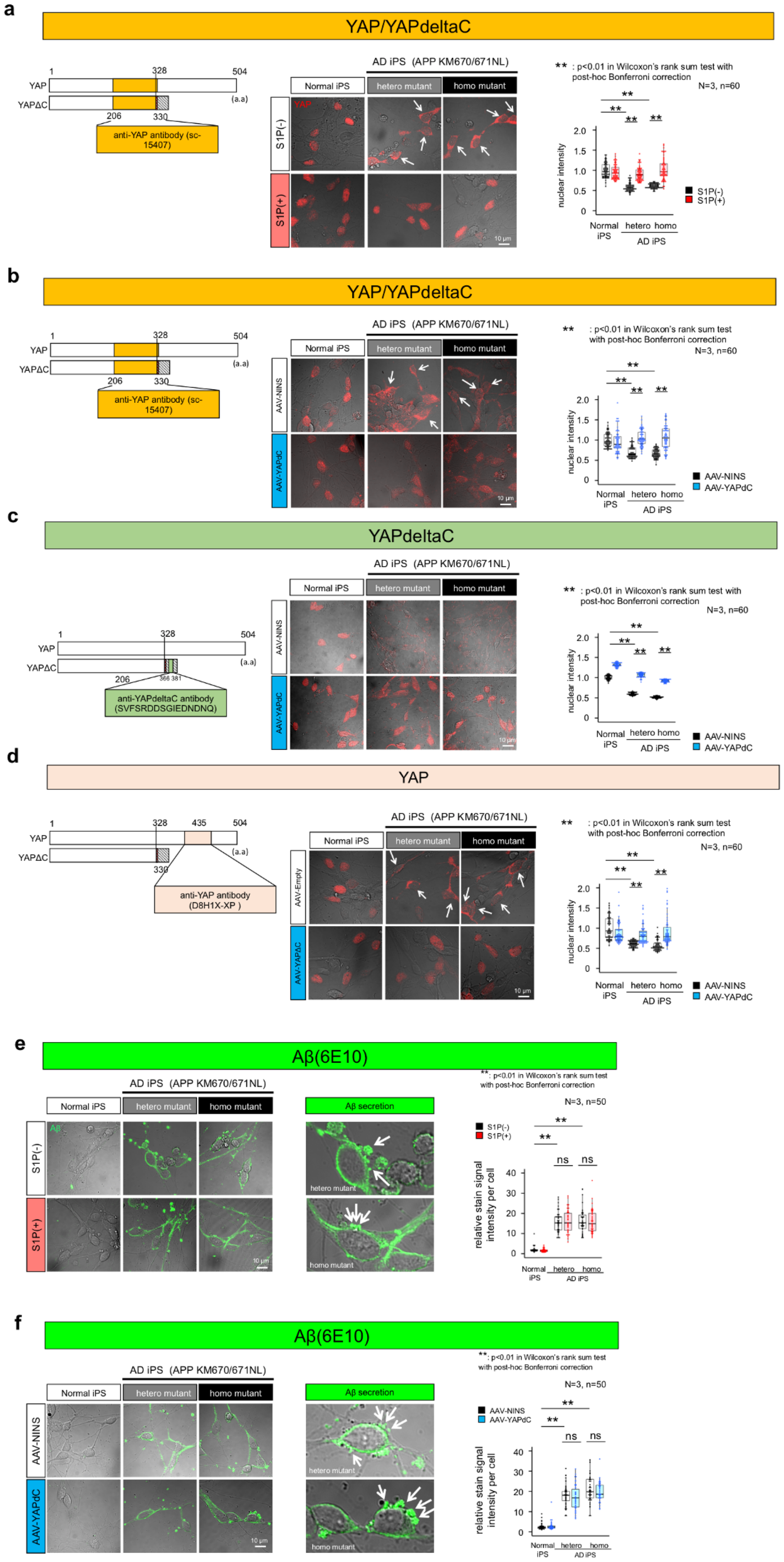

Supplementary Figure 15

Nuclear YAP recovery by S1P and YAPdeltaC without change of intracellular Aβ

a. Immunostaining of iPSC-derived AD neurons (heterozygous and homozygous mutants carrying APP KM670/671NL) after S1P treatment (corresponding to Figure 10a–e) with an anti-YAP antibody

(sc-15407) reactive both to full-length YAP and YAPdeltaC. Cells with cytoplasmic shift of YAP (white arrows) were increased in iPSC-derived AD neurons, whereas the nuclear defect of YAP/YAPdeltaC in these cells was obviously rescued by S1P treatment. The antigenic region of anti-YAP antibody used for this analysis is shown at right. P-values were determined by Wilcoxon's rank sum test with post-hoc Bonferroni correction, **p<0.01 (N=3 wells, n=60 cells).

b. Immunostaining of iPSC-derived AD neurons (heterozygous and homozygous mutants carrying APP KM670/671NL) after infection of AAV-CMV-YAPdeltaC (corresponding to Figure 10f–j) with the anti-YAP antibody. The nuclear defect of YAP/YAPdeltaC in iPSC-derived AD neurons was rescued after infection with AAV-CMV-YAPdeltaC, although cytoplasmic YAP/YAPdeltaC still remained (left panels). Full-length YAP–specific antibody revealed that FL-YAP in the nucleus was also rescued by AAV-CMV-YAPdeltaC (right panels), probably because overexpressed YAPdeltaC competitively inhibited the interaction between FL-YAP and Aβ. P-values were determined by Wilcoxon's rank sum test with post-hoc Bonferroni correction, **p<0.01 (N=3 wells, n=60 cells).

c. Immunostaining of iPSC-derived AD neurons (heterozygous and homozygous mutants carrying APP KM670/671NL) after infection of AAV-CMV-YAPdeltaC (corresponding to Figure 10f–j) with an original anti-YAPdeltaC-specific antibody. YAPdeltaC stains were reduced in heterozygous and homozygous iPSC-derived AD neurons, while the YAPdeltaC stains were recovered by AAV-CMV-YAPdeltaC in both types of iPSC-derived AD neurons. P-values were determined by Wilcoxon's rank sum test with post-hoc Bonferroni correction, **p<0.01 (N=3 wells, n=60 cells).

d. Immunostaining of iPSC-derived AD neurons (heterozygous and homozygous mutants carrying APP KM670/671NL) after infection of AAV-CMV-YAPdeltaC (corresponding to Figure 10f–j) with another anti-YAP antibody reactive only to full-length YAP. Unexpectedly, AAV-YAPdeltaC recovered also full-length YAP in the nucleus. It is presumably because overexpressed YAPdeltaC steals Aβ from full-length YAP and the balance between intracellular Aβ and full-length YAP is beneficially changed. P-values were determined by Wilcoxon's rank sum test with post-hoc Bonferroni correction, **p<0.01 (N=3 wells, n=60 cells).

e. Aβ staining before and after S1P treatment revealed that S1P did not affect the amount of intracellular Aβ in iPSC-derived AD neurons. P-values were determined by Wilcoxon's rank sum test with post-hoc Bonferroni correction, **p<0.01 (N=3 wells, n=50 cells).

f. Aβ staining before and after AAV-CMV-YAPdeltaC infection revealed that overexpressed YAPdeltaC did not affect the amount of intracellular Aβ in iPSC-derived AD neurons. Right graphs show intracellular Aβ, semi-quantified based on the signal intensity of Aβ staining per cell. No definite effect on intracellular Aβ was confirmed by S1P treatment or AAV-YAPdeltaC infection, supporting that their therapeutic effects were mediated by increase of YAP/YAPdeltaC downstream of intracellular Aβ accumulation. P-values were determined by Wilcoxon's rank sum test with post-hoc Bonferroni correction, **p<0.01 (N=3 wells, n=50 cells).

Box plots show the median, quartiles and whiskers that represent 1.5× the interquartile range. Source data are provided as a "Source Data file".

Supplementary Figure 16

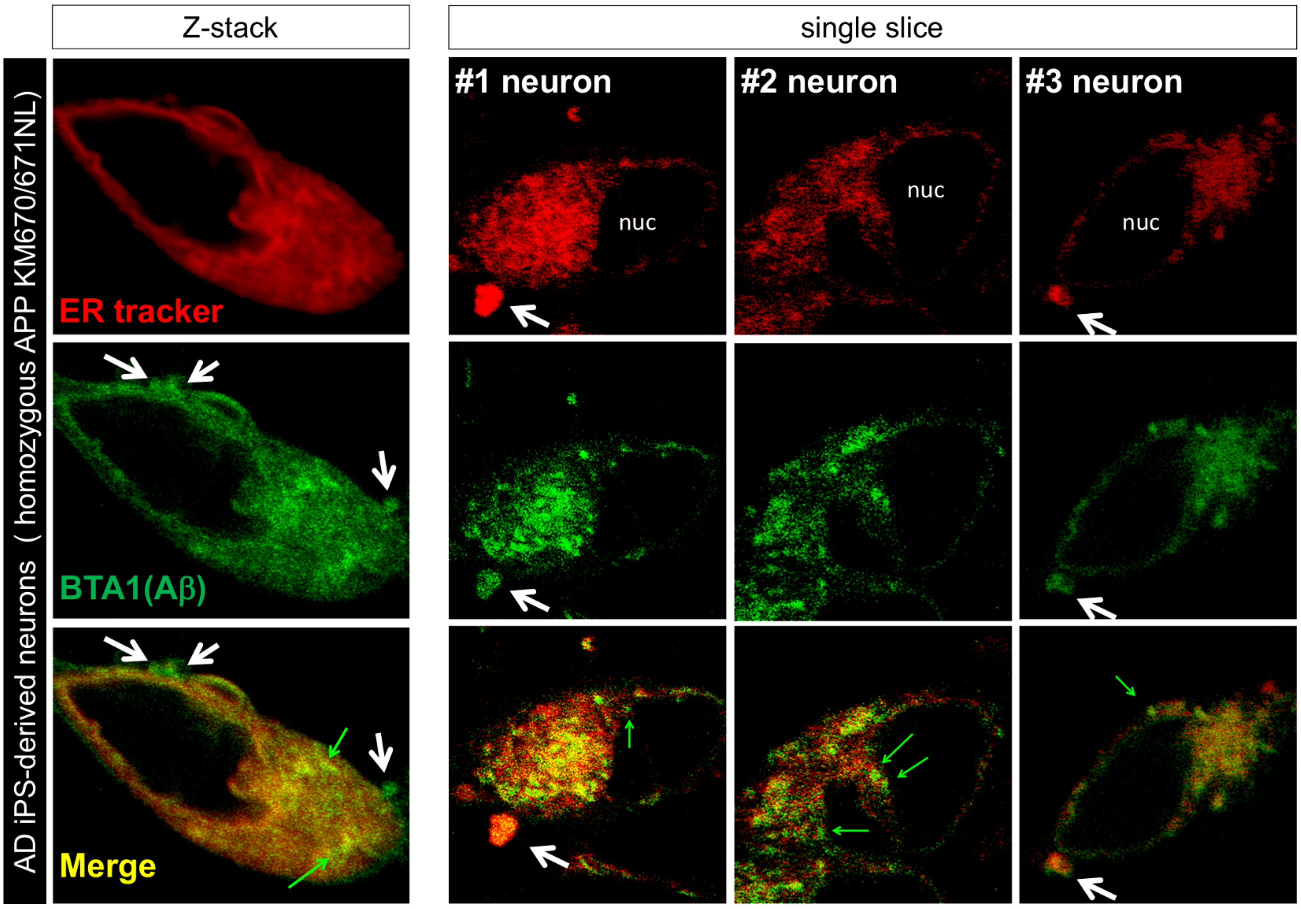

Supplementary Figure 16

High-resolution images of intracellular Aβ and ER

High-resolution images by confocal microscopy (FV1200IXGP44, Olympus, Tokyo, Japan) using the FV1200-GaAsP high-sensitivity detector revealed that a portion of Aβ shifted from the ER to cytosol (white arrow). Aβ was excreted from cell membrane by vesicles (green arrow, in which ER component was also included (red arrow)).

Supplementary Figure 17

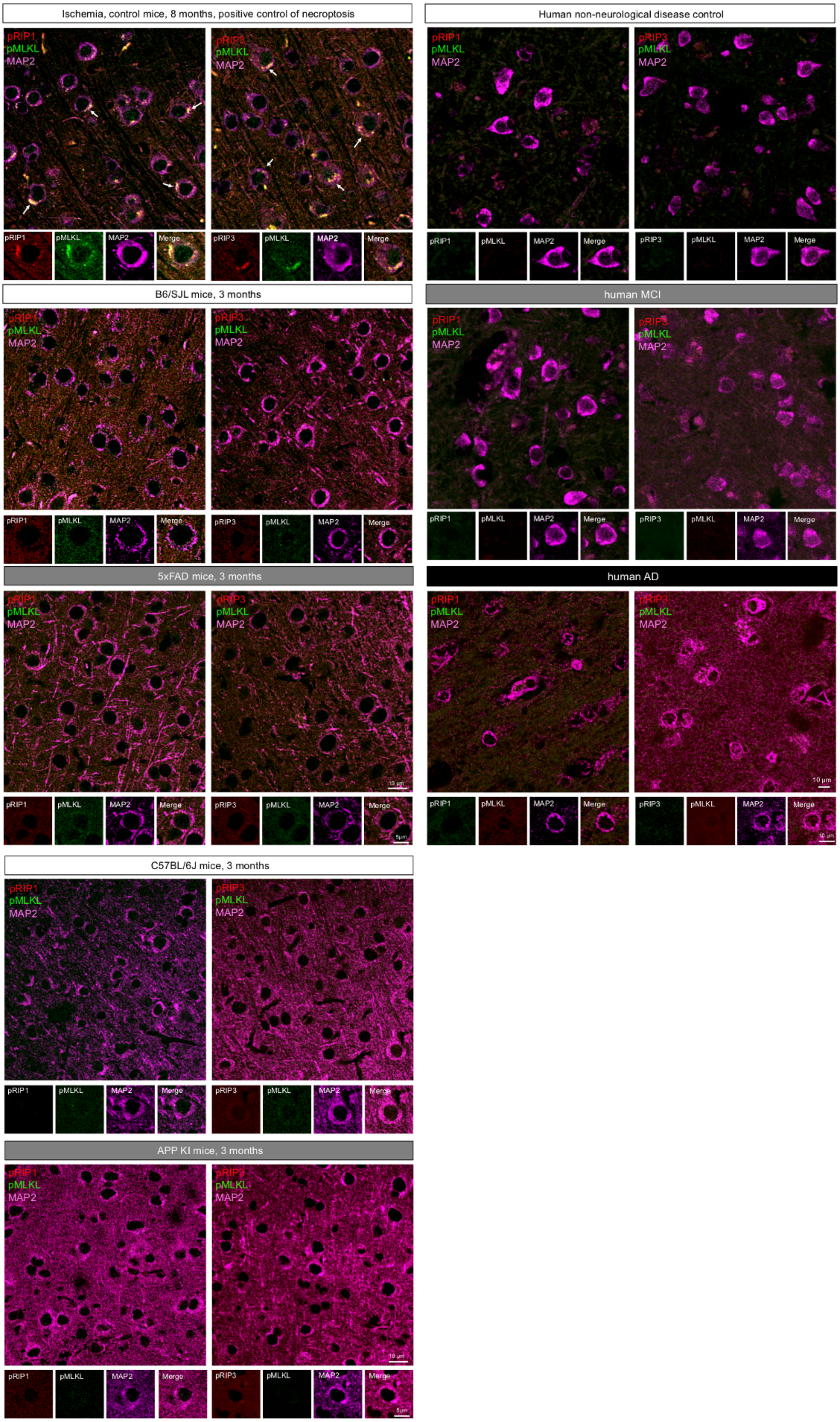

Supplementary Figure 17

Examination of necroptosis in the brains of AD model mice and AD human patients

Co-staining of phosphorylated RIP1/phosphorylated MLKL or phosphorylated RIP3/phosphorylated MLKL in B6/SJL vs. 5xFAD mice, B6 vs. APP-KI mice, and in non-neurological disease control, MCI or AD human patients. As positive control, mice after the treatment of bi-carotid artery occlusion were used. All the mouse and human tissue samples were immuno-stained simultaneously. Co-stains of phosphorylated RIP1/phosphorylated MLKL or phosphorylated RIP3/phosphorylated MLKL were observed at a single cell level in multiple neurons, while such stains were not detected in 5xFAD or APP-KI mice and in MCI or AD patients.

Supplementary Figure 18

IUPred2A https://iupred2a.elte.hu
RONN https://www.strubi.ox.ac.uk/RONN

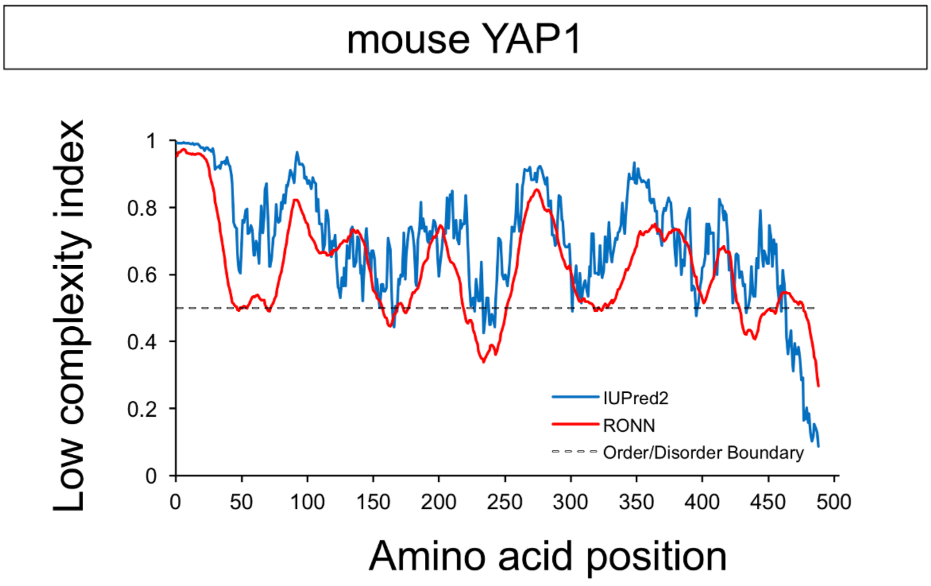

transcriptional coactivator YAP1 isoform 1 [Mus musculus]
NCBI Reference Sequence: NP_001164618.1 (488aa)

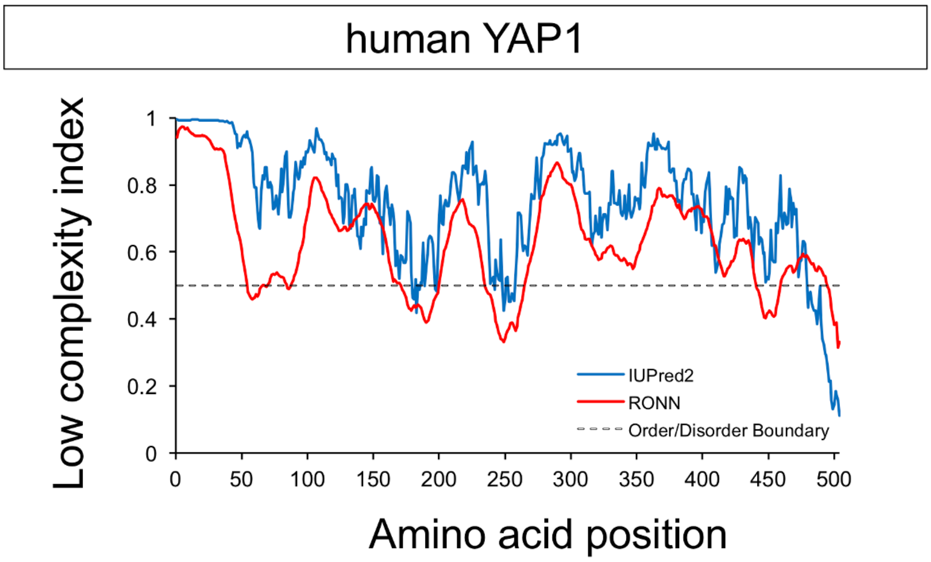

transcriptional coactivator YAP1 isoform 1 [Homo sapiens]
NCBI Reference Sequence: NP_001123617.1

Supplementary Figure 18

Distribution of low-complexity sequences in mouse and human YAP

Low-complexity score > 0.5 indicates that a region is prone to be intrinsically denatured. Low-complexity sequences are distributed throughout the mouse and human YAP proteins.

Supplementary Figure 19

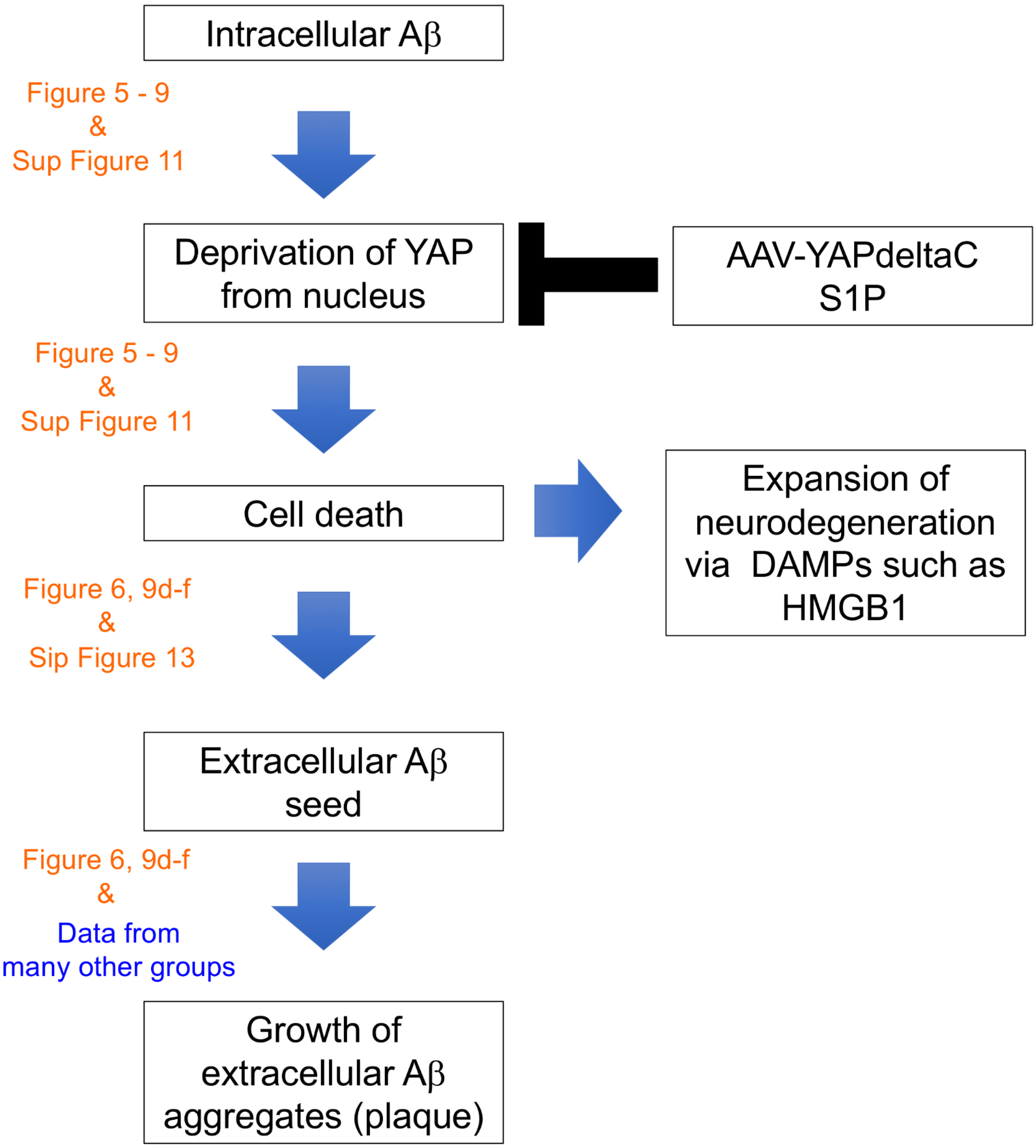

Supplementary Figure 19

Hypothesis of pathological cascade triggered by intracellular Aβ

The results in indicated figures support each step of the hypothetical pathological cascade.

Supplementary Table 1
Summary of patient information

Baseline diagnosis groups	Normal controls	Neurological disease controls		MCI		AD	
Number of patients	N=34	N=14	p-value	N=26	p-value	N=73	p-value
Age, years (S.D.)	72.71 (7.57)	70.5 (10.7)	0.715*	74.73 (6.8)	0.283*	74.62 (8.39)	0.178*
Gender			1#		0.603#		0.216#
Female (%)	13 (38.24%)	6 (42.86%)		12 (46.15%)		38 (52.05%)	
Male (%)	21 (61.76%)	8 (57.14%)		14 (53.85%)		35 (47.95%)	
ApoE4 allele number			1#		0.466#		0.389#
0	3	1		7		5	
1	1	0		9		12	
2	0	0		3		1	
MMSE (S.D.)	28.26 (1.64)	26.82 (2.32)	0.048*	22.13 (3.65)	8.4×10^{-10}*	18.18 (7.02)	3.1×10^{-15}*
CDR (S.D.)	0 (0)	0 (0)		0.5 (0)		1.5 (0.707)	
CSF Aβ42, pg/ml (S.D.)	1325 (487.57)	425.48 (209.98)	0.004*	589.59 (241.59)	0.002*	484.54 (182.62)	2.4×10^{-4}*
CSF Aβ40, pg/ml (S.D.)	NA	3081.69 (1215.41)	NA	1076.07 (2482.41)	NA	2091.76 (2485.74)	NA
CSF Tau, pg/ml (S.D.)	NA	164.45 (119.25)	NA	523.53 (289.65)	NA	521.41 (282.19)	NA
CSF pTau, pg/ml (S.D.)	55.1 (5.14)	NA	NA	77.56 (38.49)	0.265*	79.78 (39.96)	0.307*

Abbreviations: MCI, mild cognitive impairment; AD, Alzheimer's disease; ApoE, apolipoprotein E; MMSE, Mini-Mental State Examination; CDR, Clinical Dementia Rating; Aβ, amyloid β; NA, not applicable.

Statistical tests used are shown as superscript of the P values.
* Wilcoxon's rank sum test (standard deviation in parenthesis).
# Fisher's exact test (percentage in parenthesis).

Supplementary Table 2

Information about normal and disease controls

Normal control group

Patient ID	Clinical diagnosis	Sex	Age	Tau (pg/ml)	Aβ40 (pg/ml)	Aβ42 (pg/ml)	Tau/Aβ42	APOE genotype	MMSE	FAB	ALSFRS-R	ADAS
NC01	normal control	M	74						30			6.6
NC02	normal control	M	55						28			
NC03	normal control	F	78						28			8.3
NC04	normal control	F	69						30			5
NC05	normal control	F	79						30			7.7
NC06	normal control	M	76						26			
NC07	normal control	M	75						29			5.4
NC08	normal control	F	76						26			13
NC09	normal control	M	83						29			
NC10	normal control	M	76						27			5.3
NC11	normal control	F	81						30			3
NC12	normal control	M	66						30			8
NC13	normal control	M	58						29			7
NC14	normal control	M	81						29			
NC15	normal control	F	68						30			7
NC16	normal control	F	81						30			7.7
NC17	normal control	M	66						26			7
NC18	normal control	F	65						28			4.7
NC19	normal control	M	76						28			9.3
NC20	normal control	M	74						28			11
NC21	normal control	F	72						29			8
NC22	normal control	M	79						28			13.3
NC23	normal control	F	71						28			5.7
NC24	normal control	F	73						25			8.6
NC25	normal control	M	57						30			
NC26	normal control	M	59						29			
NC27	normal control	M	77						29			
NC28	normal control	M	85						26			
NC29	normal control	F	73						30			
NC30	normal control	M	74						30			
NC44	normal control	M	72	60.8		1042		E3/3	27			
NC45	normal control	M	70	53.7		1888		E3/3	24			
NC47	normal control	M	82					E3/3	28			
NC48	normal control	F	71	50.8		1045		E4/3	27			

Disease control group

Patient ID	Clinical diagnosis	Sex	Age	Tau (pg/ml)	Aβ40 (pg/ml)	Aβ42 (pg/ml)	Tau/Aβ42	APOE genotype	MMSE	FAB	ALSFRS-R	ADAS
DC31	Spinal conus syndrome	F	65	24.82	1237.875	135.739						
DC32	Neuromyelitis optica	F	62		0	0						
DC33	Paraneoplastic syndrome	F	84	143.09	4326.782	159.122			27			
DC34	Idiopathic normal pressure hydrocephalus	M	77	383.68	3621.485	202.1865			24			
DC35	Brain stem tumor	F	67	220.18	3082.283	346.5926			30			
DC36	s/o Idiopathic normal pressure hydrocephalus, alcoholism	M	60	19.26	2075.711	276.3983			27			
DC37	Peripheral neuropathy	M	67	372.95	4146.009	567.0161			25			
DC38	Idiopathic normal pressure hydrocephalus	M	75	213.51	0	419.4502			28			
DC39	s/o Idiopathic normal pressure hydrocephalus	M	80	85.87691		674.1908			27			
DC40	s/o Multiple sclerosis	F	61	186.8199		527.0526			29			
DC41	Idiopathic normal pressure hydrocephalus	M	83	98.97265		441.9792			22			
DC42	Systemic lupus erythematosus	F	48	143.1253		551.9461						
DC43	Lung cancer, Idiopathic normal pressure hydrocephalus	M	76	81.13172		804.0432			28			
DC46	Herpes zoster meningitis	M	82					E3/3	28			

MCI

Patient ID	Clinical diagnosis	Sex	Age	Tau (pg/ml)	Aβ40 (pg/ml)	Aβ42 (pg/ml)	Tau/Aβ42	APOE genotype	MMSE	FAB	ALSFRS-R	ADAS
MCI01	MCI	M	76	549.3243		381.9142	1.438345		29			
MCI02	MCI	F	51	191.0871		640.3745	0.298399		27			
MCI03	MCI	F	78	652.94		931.304	0.701103		27			
MCI04	MCI	M	72	405.3588		244.4152	1.658485					
MCI05	MCI	M	84	152.5155		251.3871	0.606696					
MCI06	MCI	F	69	886.5	875.7223	152.1252	5.827438					
MCI07	MCI	F	70	827	6656.788	224.8022	3.67879		20			
MCI08	MCI	M	72			755		E4/3	22			
MCI09	MCI	M	75			513		E3/2	22			
MCI10	MCI	F	77			835		E4/3	19			
MCI11	MCI	M	79			716		E4/3	20			
MCI12	MCI	F	70			323		E3/3	23			
MCI13	MCI	M	68			541		E4/4	19			
MCI14	MCI	M	77			879		E3/3	21			
MCI15	MCI	F	83			498		E4/3	20			
MCI16	MCI	M	73			523		E4/3	28			
MCI17	MCI	F	79			738		E4/3	14			
MCI18	MCI	M	81			550		E4/3	26			
MCI19	MCI	M	79			579		E3/3	20			
MCI20	MCI	F	71			811		E4/4	20			
MCI21	MCI	F	78			605		E4/3	20			
MCI22	MCI	M	74			748		E4/3	22			
MCI23	MCI	M	81			1124		E3/3	25			
MCI24	MCI	F	67			514		E4/4	25			
MCI25	MCI	F	77			442		E3/3	18			
MCI26	MCI	M	82			809		E3/3	22			

AD group

Patient ID	Clinical diagnosis	Sex	Age	Tau (pg/ml)	Aβ40 (pg/ml)	Aβ42 (pg/ml)	Tau/Aβ42	APOE genotype	MMSE	FAB	ALSFRS-R	ADAS
AD01	AD	M	81						26			14.7
AD02	AD	M	77						25			12.3
AD03	AD	M	79						19			19
AD04	AD	F	71						27			10.7
AD05	AD	F	72						23			9.6
AD06	AD	F	73						22			11
AD07	AD	M	81						25			17
AD08	AD	M	82						23			11.3
AD09	AD	F	79						22			11.4
AD10	AD	F	85						23			18
AD11	AD	F	69						21			7.3
AD12	AD	M	78						18			25.7
AD13	AD	F	83						20			18.3
AD14	AD	M	80						24			12
AD15	AD	F	79						20			22.4
AD16	AD	M	70						25			7
AD17	AD	M	76						19			17.4
AD18	AD	F	64						27			17.4
AD19	AD	M	82						26			20.6
AD20	AD	F	83						20			26
AD21	AD	M	80						25	12		
AD22	AD	F	75						25	11		
AD23	AD	F	73						10	9		
AD24	AD	F	57						18	7		
AD25	AD	F	59						4	6		
AD26	AD	F	62						22	8		
AD27	AD	F	61						8	8		
AD28	AD	M	54						25	13		
AD29	AD	M	82			639		E4/3	19			
AD30	AD	F	76			580		E4/3	4			
AD31	AD	M	87			838		E4/3	12			
AD32	AD	M	86			544		E4/3	10			
AD33	AD	F	73			357		E4/3	10			
AD34	AD	M	86			501		E3/3	17			
AD35	AD	F	88			574		E3/3	20			
AD36	AD	M	77			514		E4/3	18			
AD37	AD	F	81			333		E3/3	0			
AD38	AD	M	75			566		E3/3	16			
AD39	AD	F	84			533		E4/3	15			
AD40	AD	F	72			436		E4/3	0			
AD41	AD	M	69			509		E4/4	9			
AD42	AD	F	72			808		E4/3				
AD43	AD	F	86			535		E3/3	6			
AD44	AD	F	82						21			18.3
AD45	AD	M	85						15			17.3
AD46	AD	M	73						20			21
AD47	AD	M	79						21			24.7
AD48	AD	F	63						23			14
AD49	AD	F	70						24			13
AD50	AD	M	75						25			8.3
AD51	AD	F	65						25			13.7
AD52	AD	M	78			449		E4/3	23			
AD53	AD	M	77			610		E4/3	2			
AD54	AD	F	73			750		E4/3	16			
AD55	AD	M	82	277.27	5433.721	616.4906	0.449755					
AD56	AD	F	76	452.3	1861.078	225.9758	2.001541		22			
AD57	AD	M	76	1122.3	4583.199	240.5564	4.665434					
AD58	AD	F	62	305.4	3415.642	144.8123	2.108936		26			
AD59	AD	F	78						24			21
AD60	AD	M	74	713.78	3331.771	342.0334	2.086872					
AD61	AD	M	69	500.3	2065.189	209.9508	2.382939					
AD62	AD	F	76						17			9.7
AD63	AD	M	66	98.9	1789.981	117.818	0.83943		20			
AD64	AD	F	57	824.2	7858.769	327.7237	2.514924		19			
AD65	AD	F	88	863.69	5220.56	526.66	1.639938		17			
AD66	AD	M	80	575.99		411.9116	1.398334					
AD67	AD	M	74	593.6		662.9859	0.895343		15			
AD68	AD	M	66	608.33		596.9897	1.018996		11			
AD69	AD	F	74	826.9		340.8146	2.426246		6			
AD70	AD	F	56	249.7538		726.9668	0.343556		20			
AD71	AD	F	59	240.3014		280.3063	0.857281		23			
AD72	AD	M	81	200.5457		657.3948	0.305061					
AD73	AD	M	74	410.3603		453.6634	0.904548		17			

Supplementary Table 3

Primers and Oligonucleotides list

primer name	sequence
iPS isolation Fw	5’-GCATGTATTTAAAGGCAGCAGAAGC-3’
iPS isolation Rv	5’-CAATGCTTGCCTATAGGATTACCATGAAAACATG-3’

guideRNA name	sequence
gRNA for editing	5′-GGAGATCTCTGAAGTGAAGATGG-3′

oligonucleotide name	sequence
human APP KM670/671NL	5'-TTGGTTGTCCTGCATACTTTAATTATGATGTAATACAGGTTCTGGGTTGACAAATATCAAGACGGAGGAGATCTCTGAAGTGAATCTGGATGCAGAATTCCGACATGACTCAGGATATGAAGTTCATCATCAAAAATTGGTACGTAAAATAATTTACCTCTTTC-3′
