# Supplementary material for: YAP-dependent necrosis occurs in early stages of Alzheimer’s disease and regulates mouse model pathology
Source: Nat Commun. 2020 Jan 24;11:507. doi: 10.1038/s41467-020-14353-6 (PMC6981281; doi:10.1038/s41467-020-14353-6)
Supplement: Supplementary file 3 — Description of Additional Supplementary Files [file 41467_2020_14353_MOESM3_ESM.pdf]

Description of Additional Supplementary Files

Supplementary Movie 1-3

ER ballooning in genome-edited iPSC–derived homozygous AD mutant neurons

Red: ER-tracker, staining the ER. Mutant AD-iPSCs carry the APP KM670/671NL mutation, which was made by genome editing from normal iPSCs. Therefore, the backgrounds of the three lines of iPS cells were identical.

(1) Normal iPSC–derived neurons
(2) Heterozygous mutant AD-iPSC–derived neurons
(3) Homozygous mutant AD-iPSC–derived neurons

Supplementary Movie 4-6

Chronological and spatial relationships between intracellular Aβ and YAP

Red: NucRed, staining of the nucleus; green: EGFP-YAPdeltaC, staining of YAPdeltaC; blue: BTA1, staining of intracellular Aβ.

(4) Homozygous mutant AD-iPSC–derived neurons
(5) Heterozygous mutant AD-iPSC–derived neurons
(6) Heterozygous mutant AD-iPSC–derived neurons

Supplementary Movie 7-18

Necrosis after ER ballooning in genome-edited iPSC–derived homozygous AD mutant neurons

Red: ER-tracker, staining the ER; green: BTA1, staining intracellular Aβ. Mutant AD-iPSCs carry the APP KM670/671NL mutation, which was made by genome editing from normal iPSCs. Therefore, the backgrounds of the three lines of iPS cells were identical.

(7) Normal iPSC–derived neurons before treatment
(8) Heterozygous mutant AD-iPSC–derived neurons before treatment
(9) Homozygous mutant AD-iPSC–derived neurons before treatment
(10) Normal iPSC–derived neurons after S1P treatment
(11) Heterozygous mutant AD-iPSC–derived neurons after S1P treatment
(12) Homozygous mutant AD-iPSC–derived neurons after S1P treatment
(13) Normal iPSC–derived neurons after AAV-YAPdeltaC infection
(14) Heterozygous mutant AD-iPSC–derived neurons after AAV-YAPdeltaC infection
(15) Homozygous mutant AD-iPSC–derived neurons after AAV-YAPdeltaC infection
(16) Normal iPSC–derived neurons after AAV-NINS infection (negative control)
(17) Heterozygous mutant AD-iPSC–derived neurons after AAV-NINS infection (negative control)
(18) Homozygous mutant AD-iPSC–derived neurons after AAV-NINS infection (negative control)
