# Supplementary material for: YAP-dependent necrosis occurs in early stages of Alzheimer’s disease and regulates mouse model pathology
Source: Nat Commun. 2020 Jan 24;11:507. doi: 10.1038/s41467-020-14353-6 (PMC6981281; doi:10.1038/s41467-020-14353-6)
Supplement: Supplementary file 22 — Reporting Summary [file 41467_2020_14353_MOESM22_ESM.pdf]

nature research

Corresponding author(s): Hitoshi Okazawa

Last updated by author(s): Dec 14, 2019

# Reporting Summary

Nature Research wishes to improve the reproducibility of the work that we publish. This form provides structure for consistency and transparency in reporting. For further information on Nature Research policies, see Authors & Referees and the Editorial Policy Checklist.

## Statistics

For all statistical analyses, confirm that the following items are present in the figure legend, table legend, main text, or Methods section.

| n/a | Confirmed | |
|---|---|---|
| ☐ | ☒ | The exact sample size (*n*) for each experimental group/condition, given as a discrete number and unit of measurement |
| ☐ | ☒ | A statement on whether measurements were taken from distinct samples or whether the same sample was measured repeatedly |
| ☐ | ☒ | The statistical test(s) used AND whether they are one- or two-sided<br>*Only common tests should be described solely by name; describe more complex techniques in the Methods section.* |
| ☐ | ☒ | A description of all covariates tested |
| ☐ | ☒ | A description of any assumptions or corrections, such as tests of normality and adjustment for multiple comparisons |
| ☐ | ☒ | A full description of the statistical parameters including central tendency (e.g. means) or other basic estimates (e.g. regression coefficient) AND variation (e.g. standard deviation) or associated estimates of uncertainty (e.g. confidence intervals) |
| ☐ | ☒ | For null hypothesis testing, the test statistic (e.g. *F*, *t*, *r*) with confidence intervals, effect sizes, degrees of freedom and *P* value noted<br>*Give P values as exact values whenever suitable.* |
| ☒ | ☐ | For Bayesian analysis, information on the choice of priors and Markov chain Monte Carlo settings |
| ☒ | ☐ | For hierarchical and complex designs, identification of the appropriate level for tests and full reporting of outcomes |
| ☒ | ☐ | Estimates of effect sizes (e.g. Cohen's *d*, Pearson's *r*), indicating how they were calculated |

*Our web collection on statistics for biologists contains articles on many of the points above.*

## Software and code

Policy information about availability of computer code

| | |
|---|---|
| Data collection | No software was used to collect data in this manuscript. |
| Data analysis | We used a custom code for simulating the number of active cell death under an R programming environment (version 3.4.0).<br>The program code is available from our Web site ( http://suppl.atgc.info/041/ ). |

For manuscripts utilizing custom algorithms or software that are central to the research but not yet described in published literature, software must be made available to editors/reviewers. We strongly encourage code deposition in a community repository (e.g. GitHub). See the Nature Research guidelines for submitting code & software for further information.

## Data

Policy information about availability of data

All manuscripts must include a data availability statement. This statement should provide the following information, where applicable:

- Accession codes, unique identifiers, or web links for publicly available datasets
- A list of figures that have associated raw data
- A description of any restrictions on data availability

The authors declare that the data supporting the findings of this study are available within the article and supplementary information. Full anonymized data will be shared by request from any qualified investigator ("Data availability" section).

# Field-specific reporting

Please select the one below that is the best fit for your research. If you are not sure, read the appropriate sections before making your selection.

☒ Life sciences ☐ Behavioural & social sciences ☐ Ecological, evolutionary & environmental sciences

For a reference copy of the document with all sections, see nature.com/documents/nr-reporting-summary-flat.pdf

# Life sciences study design

All studies must disclose on these points even when the disclosure is negative.

| | |
|---|---|
| Sample size | We performed power analysis to estimate the required sample size (n) for each of experiments. |
| Data exclusions | There are no exclusion criteria for all analysis. |
| Replication | Experiments were independently repeated, the numbers of biological replicates are presented in the Figures. |
| Randomization | The selection of animals and the behavior analyses were performed by independent researchers.<br>Randomization (selection) of animals was simply dependent on chronological order of the birth date of animals.<br>The selection of images from immunohistochemistry/immunocytochemisty and the actual experiments of IHC/ ICC were done by different researchers. In vivo/vitro live-cell imaging were done by different researchers.<br>Western blots are repeated until the necessary N was acquired. |
| Blinding | The information about group allocation or samples were opened to the data analyst or image acquisition researchers after finalizing results (m ake graphs etc). |

# Reporting for specific materials, systems and methods

We require information from authors about some types of materials, experimental systems and methods used in many studies. Here, indicate whether each material, system or method listed is relevant to your study. If you are not sure if a list item applies to your research, read the appropriate section before selecting a response.

## Materials & experimental systems

| n/a | Involved in the study |
|---|---|
| ☐ | ☒ Antibodies |
| ☒ | ☐ Eukaryotic cell lines |
| ☒ | ☐ Palaeontology |
| ☐ | ☒ Animals and other organisms |
| ☐ | ☒ Human research participants |
| ☒ | ☐ Clinical data |

## Methods

| n/a | Involved in the study |
|---|---|
| ☒ | ☐ ChIP-seq |
| ☒ | ☐ Flow cytometry |
| ☒ | ☐ MRI-based neuroimaging |

## Antibodies

| | |
|---|---|
| Antibodies used | All antibodies used in the study are listed in the method.<br>Antibodies used for immunohistochemistry included rabbit anti-pSer46-MARCKS, 1:2000 (ordered from GL Biochem Ltd., Shanghai, China); mouse anti-amyloid beta, 1:5000 or 1:1000 (clone 82E1, #10323, IBL, Gunma, Japan); rabbit anti-pSer909-LATS1, 1:100 (#9157, Cell Signaling Technology, Danvers, MA, USA); rabbit anti-pThr210-PLK1, 1:5000 (#ab155095, Abcam, Cambridge, UK); rabbit anti-YAP, 1:100 (sc-15407, Santa Cruz Biotechnology, Dallas, TX, USA); mouse anti-MAP2,1:100 (sc-32791,Santa Cruz, Dallas, TX, USA; mouse anti-RIP1, 1:200 (610459, BD bioscience, CA, USA); rabbit anti-RIP3, 1:250 (ab56164, Abcam, Cambridge, UK); rabbit anti-phosphoSer166-RIP1,1:400 (#44590, Cell Signaling Technology); rabbit anti-phosphoSer232-RIP3,1:100 (ab195117, abcam) ; mouse anti-KDEL, 1:100 (ADI-SPA-827, Enzo, NY, USA); donkey anti-mouse IgG Alexa488, 1:1000 (#A-21202, Molecular Probes, Eugene, OR, USA); donkey anti-rabbit IgG Alexa568, 1:1000 (#A-10042, Molecular Probes).<br>Antibodies used for immuno-electron microscopy included rabbit anti-pSer46-MARCKS, 1:1000 (ordered from GL Biochem Ltd., Shanghai, China); nanogold conjugated goat anti-rabbit secondary antibody 1:100 (N24916, Thermo Fisher).<br>Antibodies used for immunoprecipitation included rabbit anti-YAP (#14074, Cell Signaling Technology); mouse anti-amyloid beta, 1:5000 (clone 82E1, #10323, IBL, Gunma, Japan).<br>Antibodies used for dot blot included mouse anti-amyloid beta,1:5000 (clone 82E1, #10323, IBL, Gunma, Japan); HRP-conjugated anti-mouse IgG,1:5000 (GE Healthcare, NA931VA).<br>Antibodies used for western blotting included rabbit anti-YAP (H-125), 1:3000 (sc-15407,Santa Cruz Biotechnology); mouse anti-amyloid beta, 1:1000 (clone 82E1, #10323, IBL, Gunma, Japan); mouse anti-RIP1, 1:1000 (610459, BD bioscience, CA, USA); rabbit anti-RIP3, 1:1000 (ab56164, Abcam, Cambridge, UK); rabbit anti-phosphoSer166-RIP1, 1:1000 (#44590, Cell Signaling Technology); rabbit phosphoSer232-RIP3, 1:1000 (ab195117, abcam); HRP-conjugated anti-mouse IgG, 1:3000 (GE Healthcare, NA931VA); HRP-conjugated anti-rabbit IgG 1:3000 (NA934VS, GE Healthcare). |

| | |
|---|---|
| | Antibodies used for Immunocytochemistry included rabbit anti-YAP(D8H1X-XP), 1:100 (14074S9, Cell Signaling Technology); rabbit-anti-YAP(H-125), 1:200 (sc-1540, Santa Cruz Biotechnology); mouse anti-amylod beta, 1:250 (Covance, NJ, USA, 6E10, SIG-39300); Cy3-conjugated anti-mouse IgG, 1:500 (715-165-150, Jackson Laboratory); and Alexa Fluor 488–conjugated anti-rabbit IgG,1:1000 (A11008, Molecular Probes). |
| Validation | Information of validation of commercially available antibodies are provided on the manufacturer's websites. Rabbit anti-pSer46-MARCKS antibody was originally prepared and was validated in our previous publication (ref#18, Fujita et al., 2016, Sci Rep, doi:10.1038/srep31895) |

## Animals and other organisms

Policy information about studies involving animals; ARRIVE guidelines recommended for reporting animal research

| | |
|---|---|
| Laboratory animals | We wrote the species, strain, and sex in the method, and age of animals were indicated in the figure and figure legends ("figure legends"). |
| Wild animals | The study did not involve any wild animals. |
| Field-collected samples | The study did not involve any samples collected from the field. |
| Ethics oversight | We wrote the statement that our experiments follow ethical regulations in the "ethics" section in the method ("Ethics"). |

Note that full information on the approval of the study protocol must also be provided in the manuscript.

## Human research participants

Policy information about studies involving human research participants

| | |
|---|---|
| Population characteristics | Information of human patient' samples were described in the "Extended Data Table S1 and S2" and method. |
| Recruitment | Cohort 1 consists of four normal controls, one patient without dementia but with another neurological disease (disease control), 19 patients with MCI, and 18 patients with AD in Higashi Matsudo Municipal Hospital. Cohort 2 comprised 13 disease controls, seven MCI patients, and 17 AD patients in The University of Tokyo. Cohort 3 comprised 30 normal controls and 30 AD patients in Tohoku University. Cohort 4 comprised eight AD patients in Nagoya University. |
| Ethics oversight | Informed consent for the use of human CSF was obtained and approved by the appropriate ethics committee at each institution, Higashi Matsudo Municipal Hospital, The University of Tokyo, Tohoku University and Nagoya University. Furthermore, informed consent for the use of all human CSF was obtained and approved by the ethics committee of Tokyo Medical and Dental University. |

Note that full information on the approval of the study protocol must also be provided in the manuscript.
